# Vispro improves imaging analysis for Visium spatial transcriptomics

**DOI:** 10.1186/s13059-025-03648-w

**Published:** 2025-06-18

**Authors:** Huifang Ma, Xingyuan Zhang, Yilong Qu, Anru R. Zhang, Zhicheng Ji

**Affiliations:** 1 Department of Biostatistics and Bioinformatics, Duke University School of Medicine, Durham, NC USA; 2 Computational Biology and Bioinformatics Program, Duke University School of Medicine, Durham, NC USA; 3 Department of Computer Science, Duke University Trinity College of Arts & Sciences, Durham, NC USA

## Abstract

Spatial transcriptomics enables spatially resolved gene expression analysis, but accompanying histology images are often degraded by fiducial markers and background regions, hindering interpretation. To address this, we introduce Vispro, an end-to-end automated image processing tool optimized for 10× Visium data. Vispro includes modules for fiducial marker detection, image restoration, tissue region detection, and segmentation of disconnected tissue areas. By enhancing image quality, Vispro improves the accuracy and performance of downstream analyses, including tissue and cell segmentation, image registration, gene expression imputation guided by histological context, and spatial domain detection.

**Supplementary Information:**

The online version contains supplementary material available at 10.1186/s13059-025-03648-w.

## Background

Spatial transcriptomics (ST) is a transformative technology that simultaneously measures gene expression profiles, spatial locations, and imaging information from the same set of cells. Unlike single-cell sequencing, which cannot capture spatial or imaging data, ST provides a deeper understanding of how different cell types are spatially organized within a tissue and enables the study of cell morphology.

The histology imaging information accompanying ST has become indispensable in many analytical methods designed for ST data [1–13], with two primary approaches currently being explored. One approach [1–9] leverages image data to predict gene expression or facilitate gene analysis, aiming to extract molecular insights from histological images. A notable example is TESLA [9], which estimates tissue contours and generates superpixel regions based on histological similarity, enabling high-resolution gene imputation. The other approach [10–13] integrates both gene expression data and imaging information to develop more comprehensive models, using the spatial correlation between these two modalities to improve the accuracy and depth of biological interpretation. For example, SpaGCN [10] integrates histological images with gene expression data and spatial cell locations to infer spatial domains and identify spatially variable genes. Similarly, stLearn [11] combines imaging, gene expression, and spatial distances between cells to map spatiotemporal trajectories and cell-cell interactions. Additionally, imaging data has demonstrated significant potential in enhancing the accuracy of aligning multiple tissue sections, as highlighted by several studies [14]. This alignment facilitates integrative analysis of ST data across different samples, providing deeper insights into spatial gene expression patterns and tissue architecture.

**Fig. 1:**
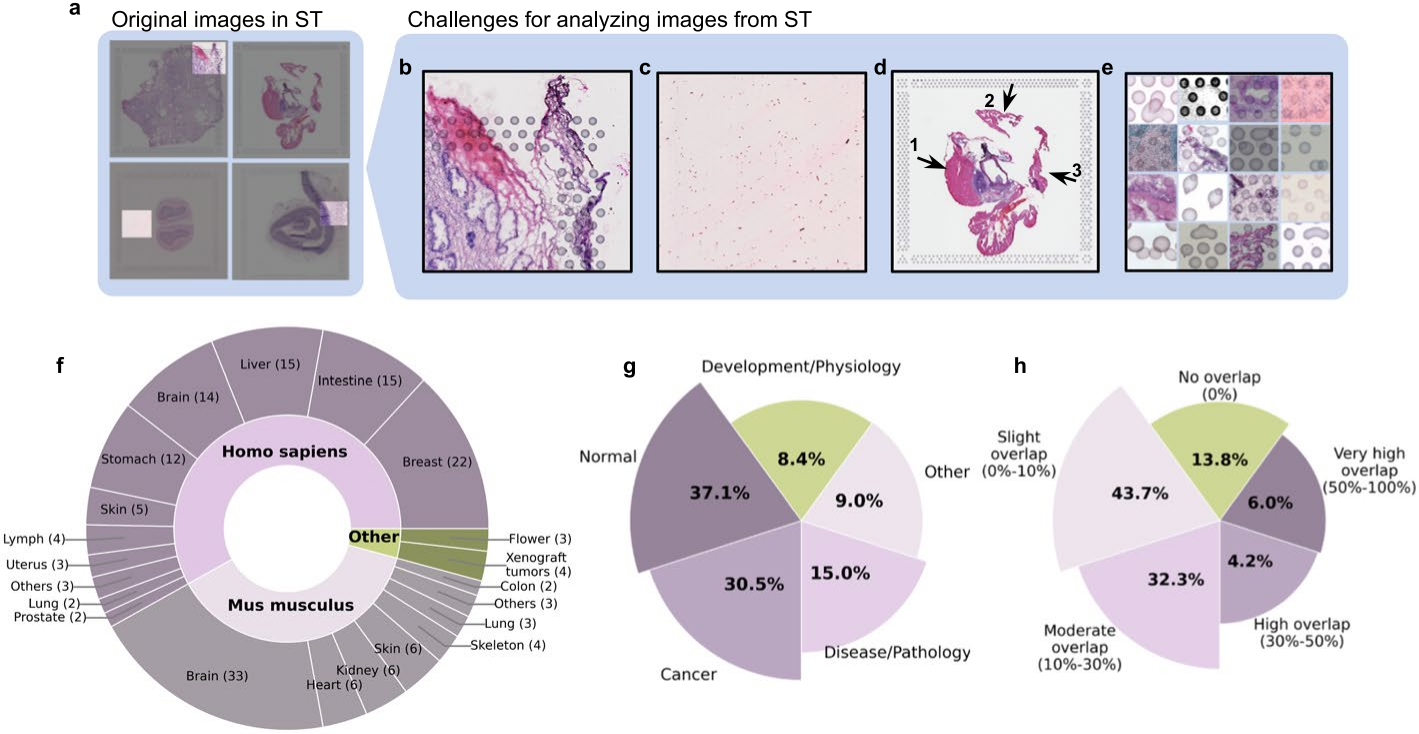
Challenges in analyzing Visium images and training data used in Vispro. **a** Original H&E-stained tissue images captured by the Visium platform. Zoomed-in views of the highlighted regions of interest are provided in **b**-**e**. **b** An example showing fiducial markers overlaying tissue regions. **c** An example showing background noise in the blank regions. **d** An example of segregated tissue regions, with three subregions marked as 1, 2, and 3. **e** Examples of different types of deformation in fiducial markers. **f** Number of images (shown in parentheses) from different tissue types (outer ring) and different species (inner ring). **g** Proportion of images (number shown within each pie) across different disease and developmental conditions. **h** Proportion of images (number shown within each pie) with different overlap proportions (indicated outside each pie) between fiducial markers and tissue regions. The overlap proportion is calculated as the proportion of all fiducial markers that overlap with tissue regions and is indicated within parentheses in the text outside each pie

Despite the promise of image-based approaches in ST, multiple challenges arise when analyzing images from ST datasets. First, images from 10× Visium, one of the most widely used ST platforms, include artificial reference points known as fiducial markers (Fig. 1a, b), which are intended to outline tissue regions. However, these markers often create challenges during image processing. They can overlap with tissue regions due to operational errors, obscuring important structures and textures. Their blob-like shape frequently leads to misclassification as tissue structures, and their consistent square arrangement can confuse models attempting to learn spatial tissue patterns, leading to misinterpretation of global tissue architecture. Second, most ST images contain both tissue regions, where cellular structures are visible, and blank regions, referred to as background (Fig. 1a, c). The background often contains residual stains and random noise, blurring tissue boundaries and interfering with gene expression predictions when entire images are used. Third, a single ST image may capture multiple tissue samples, particularly when tissue microarrays (TMAs) are used, containing tissues from different spatial locations or individuals (Fig. 1a, d). Without proper segmentation of these distinct regions, downstream analysis becomes significantly more complicated.

These challenges have been largely overlooked and underexplored in the existing literature, particularly in relation to handling fiducial markers (Additional file 1: Table S1). The 10× Genomics Space Ranger [15] and Loupe Browser [16] can automatically or manually detect fiducial markers. However, they rely on a standardized fiducial marker template for alignment, assuming all markers are perfectly circular and uniformly arranged. This assumption fails to account for the deformations or misprints commonly present in real images (Fig. 1a, e). More critically, these tools cannot automatically remove fiducial markers from images or recover distorted tissue regions where markers overlap with tissue. Additionally, existing methods [17–21] for detecting and segmenting tissue regions are ineffective when applied directly to ST images without first removing fiducial markers, leading to erroneous results, as we will demonstrate. Although manual manipulation of images can address some of these issues, it is time-consuming and impractical for large-scale datasets. In summary, there is currently no method that can automatically and efficiently process ST images to fully remove technical artifacts and produce images ready for downstream analysis.

**Fig. 2:**
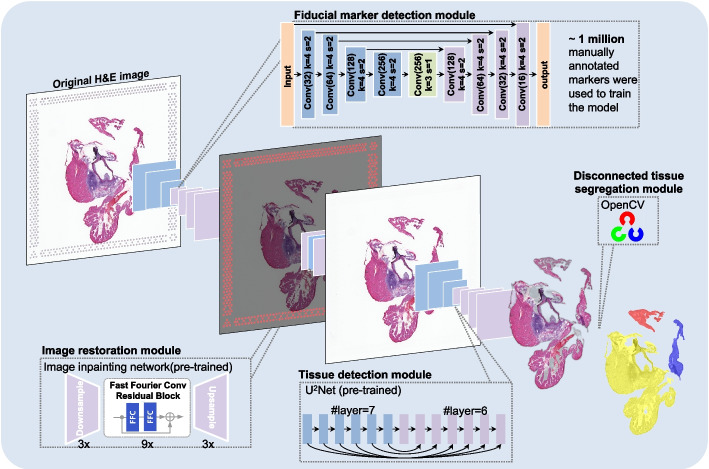
Vispro pipeline to improve the quality of Visium ST images. The complete Vispro workflow consists of four modules: marker detection, image restoration, tissue detection, and disconnected tissue segregation

To address these challenges, we developed Vispro, a fully automated image processing tool for histology images (Fig. 2). Vispro includes four sequential modules: detecting fiducial markers, removing these markers and restoring the image, detecting tissue regions, and segregating disconnected tissue areas. The tool outputs cleaned images with accurately defined tissue regions, ready for integration into other image analysis software. We demonstrate that Vispro provides higher-quality image data in ST, including more accurate fiducial marker localization and tissue boundary detection. Additionally, we show that images processed by Vispro significantly improve the performance of downstream tasks such as cell segmentation, image registration, gene imputation, and spatial domain detection. In summary, Vispro is a valuable tool for optimizing the use of image data in ST.

## Results

### Vispro overview

Vispro is built upon a series of deep-learning models and computer vision algorithms to perform image analysis tasks across its four modules (Fig. 2). The fiducial marker detection module employs a deep neural network based on U-Net [20], with customized layers and loss functions designed to address the scale disparity between tiny fiducial markers and the large whole image. By emphasizing key shallow and deep layers and incorporating focal loss to address data imbalance, Vispro achieves improved accuracy in detecting small circular targets, outperforming Hough circle transform [22], CellPose [23], CircleNet [24], and the baseline U-Net [20]. To train the model, we manually annotated approximately 100,000 fiducial markers from 167 ST images, encompassing various biological contexts and varying degrees of overlap with tissue regions (Fig. 1f–h). The fiducial marker detection network produces a binary segmentation mask for fiducial markers. In the image restoration module, both the segmentation mask and the original image are fed into a pre-trained image inpainting neural network, which restores the content in the marker area while preserving the surrounding image context. The tissue detection module utilizes a pre-trained U$^2$Net neural network to identify the salient object and separate the tissue from the background. Finally, the disconnected tissue segregation module identifies and separates disjoint tissue regions by analyzing pixel connectivity using the optimized connected-component labeling function from the OpenCV library. The complete pipeline operates in real time and in an end-to-end manner, with manageable memory requirements that enable the processing of large datasets.

### Vispro accurately identifies and removes fiducial markers

Fiducial marker removal is not a simple task, as fiducial markers exhibit various levels of overlap with tissue regions in most ST datasets (Fig. 1a). While a simple method such as image cropping can remove the fiducial markers, it also destroys parts of the tissue regions that may contain valuable information. A deep neural network, like the one employed by Vispro, is needed to effectively remove fiducial markers while preserving the integrity of the tissue regions.

**Fig. 3:**
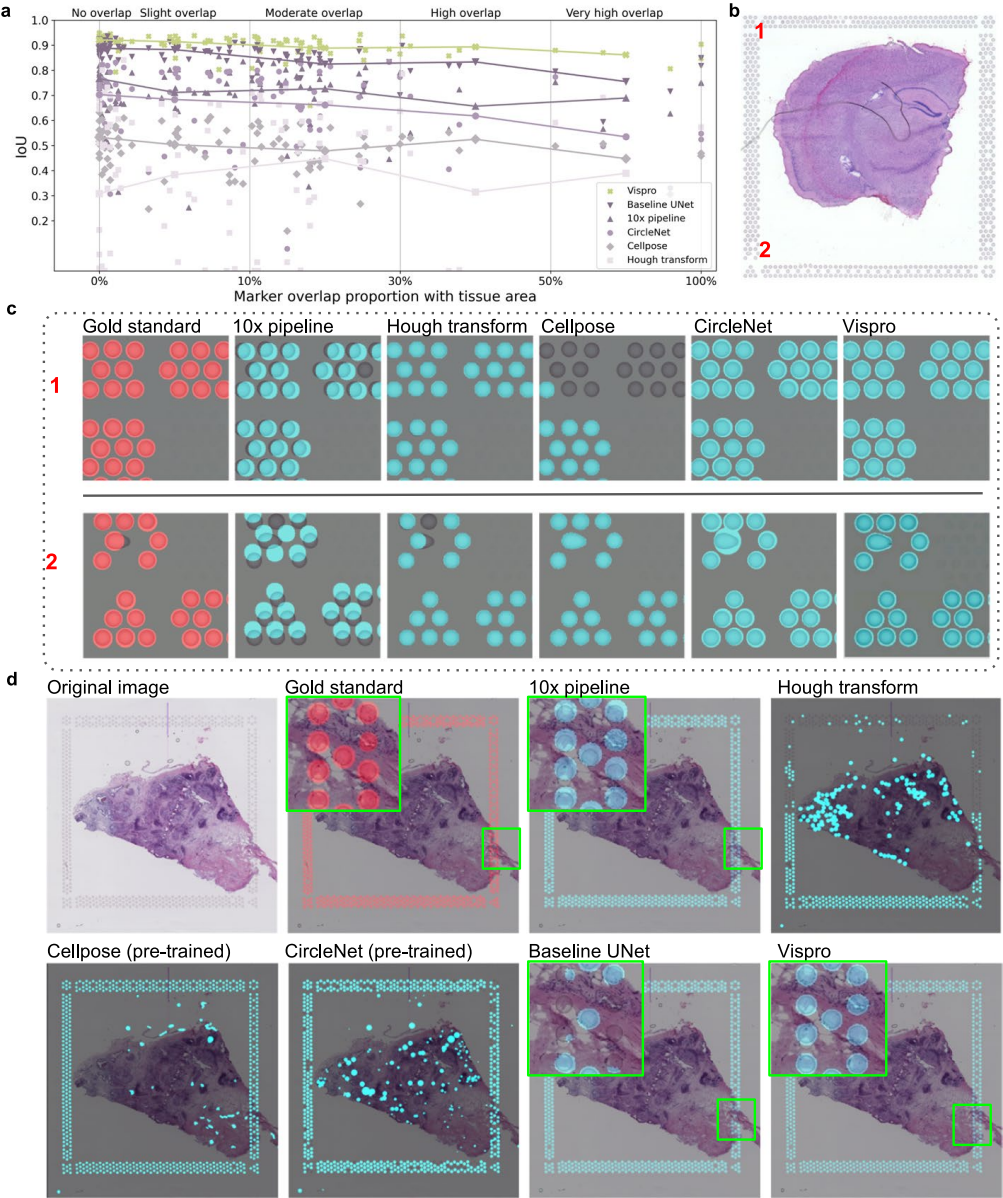
Evaluation of fiducial marker detection. **a** IoU of fiducial marker detection accuracy (*y*-axis) plotted against different overlap proportions (*x*-axis). Each point represents an image. **b**,** c** An example Visium ST image (**b**) with zoomed-in views of the gold standard marker regions (red circles) and fiducial markers detected by different methods (cyan circles), displayed from left to right in two distinct corners (**c**). **d** Visual comparison of fiducial marker detection results on a representative sample. The gold standard marker regions (red circles) is shown alongside fiducial markers detected by different methods (cyan circles). Insets highlight zoomed-in views of regions where markers overlap with tissue

We compared the performance of the 10× pipeline, Hough circle transform [22], pre-trained Cellpose [23], pre-trained CircleNet [24], the baseline U-Net architecture [20], and the fiducial marker detection module in Vispro for detecting fiducial markers in a cross-validation study (Methods). We used the Intersection over Union (IoU) metric to evaluate the performance of all six methods against the manually annotated gold standard. Vispro consistently outperforms existing methods across fiducial markers with varying levels of overlap with tissue regions (Fig. 3a). Vispro achieves an IoU improvement of over 10% compared to the 10× Genomics pipeline and approximately 5% compared to the baseline U-Net, demonstrating its superior accuracy in identifying fiducial markers compared to existing methods.

Figure 3b, c shows an example where the IoU for the 10× pipeline is below 0.3. We omitted the baseline U-Net as its performance is similar to Vispro in non-overlapping regions. In this example, the Visium slice shows global deformations, causing the fiducial marker arrangement to display shape distortions. Consequently, the 10× Genomics template struggles to align with the markers, resulting in missed fiducial markers (e.g., corner 1) and substantial displacement (e.g., corner 2). Similarly, Hough and Cellpose also result in missed fiducial markers. While CircleNet successfully recognizes all fiducial markers, the circular shapes do not align well with distorted fiducial markers that deviate from a perfect circle. In contrast, the learning-based method employed by Vispro effectively leverages texture information to accurately localize the marker areas, with an IoU of 0.94.

Figure 3d shows another example where fiducial markers overlap with tissue regions. The 10× pipeline exhibits slight spatial misalignment between the marker template and the actual fiducials. CircleNet, Cellpose, and the Hough circle transform produce numerous false positives within the tissue and frequently miss true positives. Although the baseline U-Net is trained specifically on marker data, its performance declines in overlapping regions, suggesting limited generalizability. In contrast, Vispro delivers the most consistent and visually accurate segmentations across both background and tissue-overlapping regions.

**Fig. 4:**
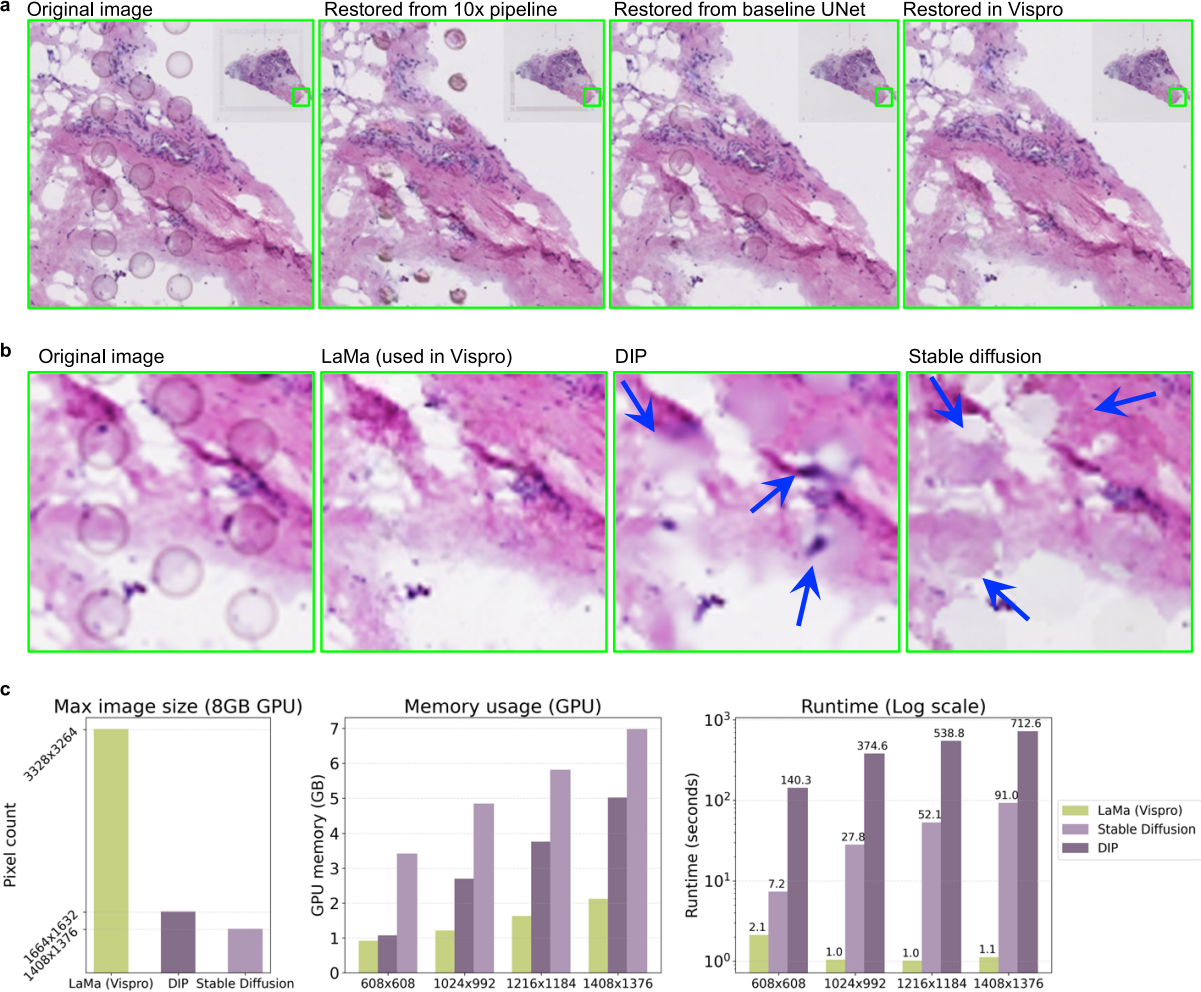
Evaluation of image restoration. **a** Visual comparison of restoration results using LaMa with masks of fiducial markers detected by the 10× pipeline, baseline U-Net, and Vispro. Insets show the images of the whole tissue. **b** Enlarged views of restoration results are shown for LaMa (Vispro), DIP, and Stable Diffusion, with all methods performed using masks of fiducial markers detected by Vispro as input. **c** Quantitative comparison of maximum image size supported with an 8 GB GPU (left), GPU memory usage for processing images of different sizes (middle), and runtime for processing images of different sizes (right) for LaMa (Vispro), DIP, and Stable Diffusion

Figure 4a illustrates the results of image restoration through inpainting the identified fiducial markers. The binary masks of fiducial markers obtained from the 10× pipeline, baseline U-Net, and Vispro were provided as input to the same inpainting algorithm, LaMa [25] (Methods). Using the fiducial markers identified by Vispro, the inpainting process successfully removes all markers from both the background region and the tissue region in the images. In contrast, noticeable fiducial markers remain in the images when using the results from the 10× pipeline, while the baseline U-Net fails to recover some fiducial markers overlapping with the tissue region.

We further compared the restoration quality of LaMa with two alternative methods, DIP [26] and Stable Diffusion [27] (Fig. 4b). All methods were applied to the fiducial markers obtained from the fiducial marker detection module of Vispro. LaMa produced consistent and high-fidelity restorations across scales. In comparison, substantial inconsistencies are observed between the real image and the images restored by DIP and Stable Diffusion, especially around the borders of the detected fiducial markers. In terms of computational efficiency, LaMa consistently maintained the lowest runtime and memory usage across all resolutions, making it suitable for processing large-scale H&E images (Fig. 4c). Thus, we selected LaMa to perform image restoration in Vispro. Additional image restoration results are provided in Additional file 2: Fig. S1.

### Vispro accurately identifies and segregates tissue regions

**Fig. 5:**
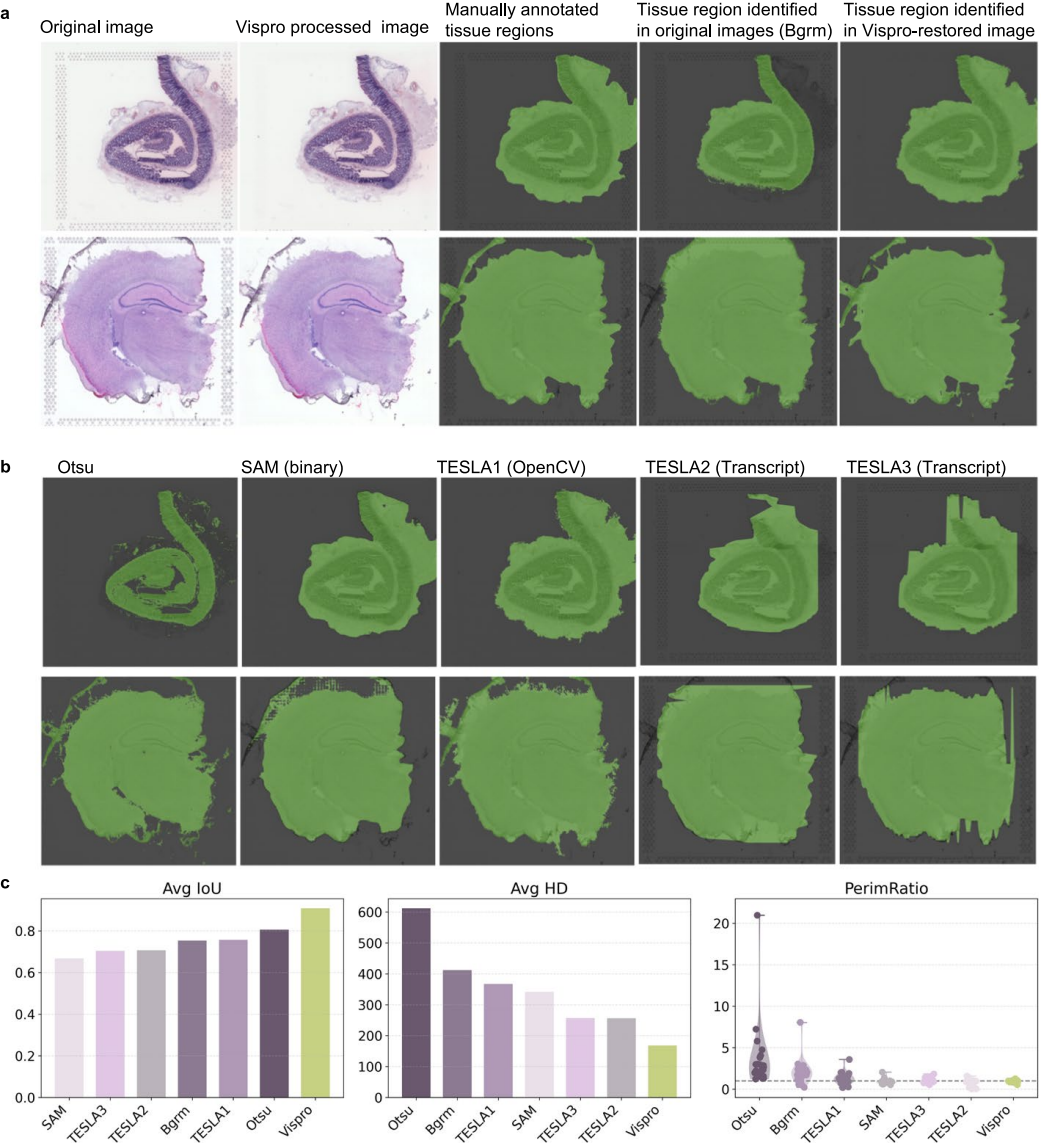
Tissue detection results. **a** From left to right: the original images, the images processed by Vispro’s fiducial marker detection and image restoration modules, the manually annotated tissue areas serving as the gold standard (green regions), the detected tissue regions from the original image (green regions), and the detected tissue regions from the Vispro-processed image in the second column. **b** Tissue regions identified by competing methods, including Otsu, SAM, OpenCV-based TESLA1, and transcript-based TESLA2 and TESLA3. All methods were applied to images processed by Vispro’s fiducial marker detection and image restoration modules. **c** Quantitative evaluation of segmentation performance. From left to right: average Intersection-over-Union (IoU), average Hausdorff distance (HD), and distribution of perimeter ratio, with the ideal value being 1. Each metric is computed against gold standard manual annotations

**Fig. 6:**
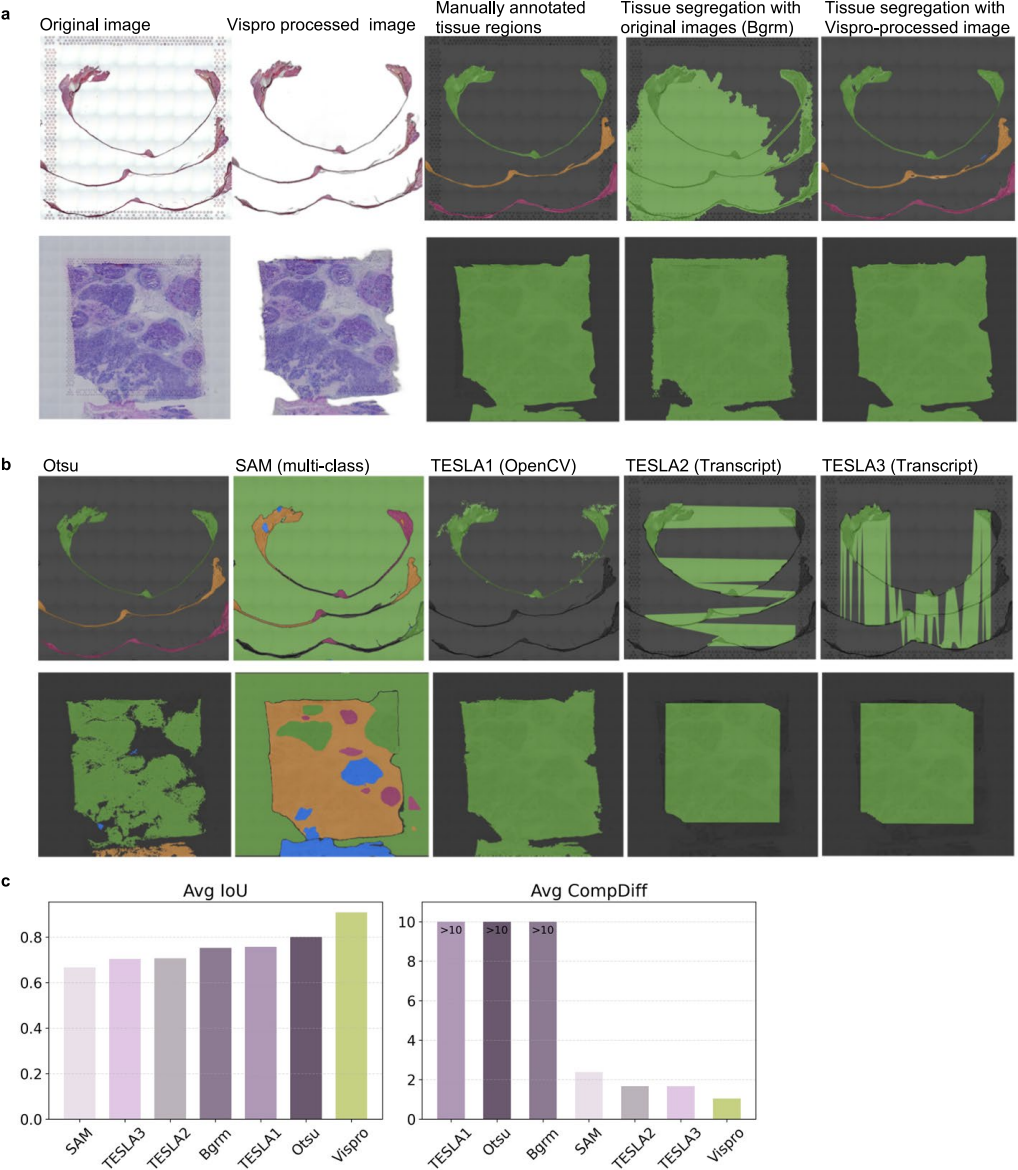
Disconnected tissue segregation results. **a** From left to right: the original images, Vispro-processed images after tissue detection, manually annotated tissue regions, tissue segregation results using the original image, and tissue segregation results using the Vispro-processed images in the second column. **b** Tissue segregation results from competing methods on images processed by Vispro’s tissue detection module. **c** Quantitative evaluation of tissue area segmentation. Left: average Intersection-over-Union (IoU) between predictions and manual annotations. Right: average component difference (CompDiff), measuring over- or under-segmentation in the number of connected components. The ideal CompDiff is 0

After inpainting the fiducial markers identified by Vispro, we evaluated Vispro’s performance in differentiating tissue regions from background regions (Fig. 5) and in a subsequent module for segregating disconnected tissue regions (Fig. 6). The computationally identified tissue regions and segregated tissue subregions were visually and quantitatively compared to the gold standard of manually annotated tissue regions.

Vispro accurately distinguishes tissue regions from background regions (Fig. 5a). Using images free of fiducial markers, the tissue regions identified by Vispro closely match the gold standard. In contrast, directly identifying tissue regions without removing or inpainting the fiducial markers results in significantly worse outcomes. The presence of fiducial markers leads to enlarged or truncated tissue regions, particularly when the markers overlap with actual tissue areas. We further compared the performance of alternative methods for identifying tissue regions, all applied to images free of fiducial markers (Fig. 5b). Otsu [17] and Segment Anything Model [18] (SAM) exhibit poor boundary localization, and Otsu tends to over-segment. The OpenCV-based method [19] implemented by TESLA provides slightly improved spatial consistency but still suffers from irregular edges. Transcript-derived segmentations implemented by TESLA are more structured, yet they show misalignment with tissue morphology. Quantitatively, Vispro achieves an average IoU close to 1, a low average Hausdorff distance, and perimeter ratios close to the ideal value of 1 (Fig. 5c). All other methods show lower average IoUs, higher average Hausdorff distances, and perimeter ratios deviating from 1. These results suggest that Vispro is the best-performing method for identifying tissue regions.

After tissue region detection, Vispro effectively identifies disconnected tissue subregions (Fig. 6a). In most cases, it accurately detects the correct number of subregions. In contrast, segmentation based solely on the original images shows a significant decline in performance, particularly by missing key tissue regions or incorrectly merging thin structures with the background region. We further compared segmentation performance by applying Vispro’s tissue segregation module to the tissue regions identified by competing methods (Fig. 6b). For SAM, we leveraged its multi-class segmentation capability for tissue segregation, which directly outputs multiple image segments. Transcript-informed models implemented by TESLA produce contiguous masks that include both tissue and background while overlooking regions lacking transcript data. The OpenCV-based method implemented by TESLA cannot detect multi-segment tissue slices. SAM identifies multiple tissue components but misses thinner structures and tends to over-segment. Otsu performs best for multi-segment slices but often over-segments due to its thresholding nature and fails to maintain spatial consistency. Quantitatively, the IoU metric of Vispro remains high (Fig. 6c), indicating that Vispro consistently predicts tissue areas with high precision. In comparison, IoU values of other methods decrease substantially. Vispro also has the smallest average component difference from the ground truth, indicating its high accuracy in identifying tissue segments. Additional tissue region identification results are provided in Additional file 2: Fig. S2 and Additional file 2: Fig. S3.

### Vispro improves cell segmentation

Cell segmentation aims to identify and separate individual cells in an image [28, 29]. Accurately identifying cell locations is crucial for understanding the spatial organization of different cell types within a tissue. Since most cell segmentation methods rely solely on imaging information, the quality of the images significantly impacts the performance of cell segmentation.

**Fig. 7:**
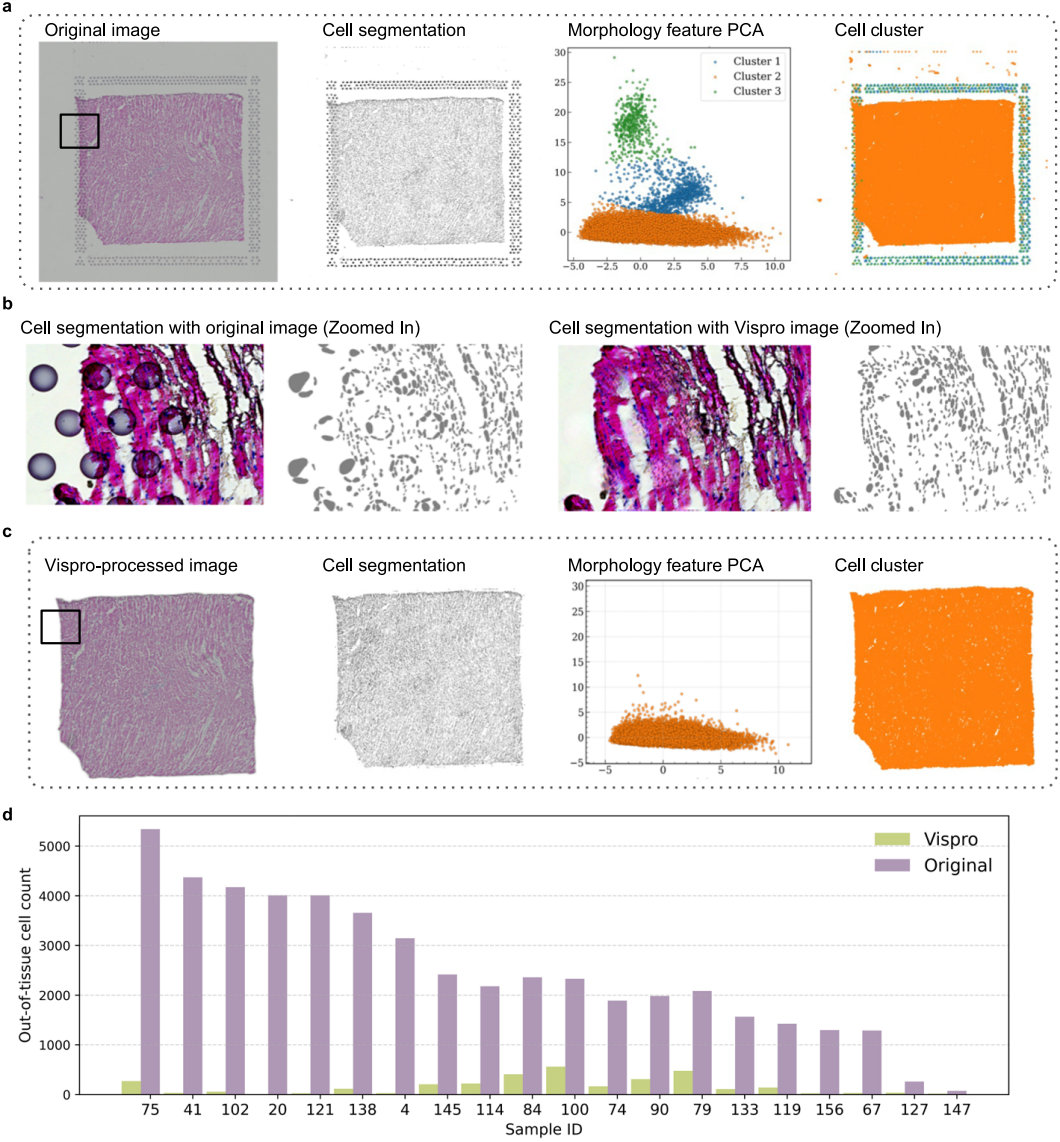
Cell segmentation results. **a** From left to right: the original image, cell segmentation results using the original image, PCA projection of cell morphological features, colored by cell clusters derived from the original image, and spatial locations of cells from different clusters shown on the original image. **b** Zoom-in comparison of cell segmentation quality between the original image (left) and Vispro-processed image (right), highlighting areas affected by fiducial markers. **c** From left to right: the Vispro-processed image, cell segmentation results with the Vispro-processed image, PCA projection of cell morphological features, colored by cell clusters derived from the Vispro-processed image, and spatial locations of cells from different clusters shown on the Vispro-processed image. **d** Quantitative evaluation of the number of segmented cells outside tissue regions

We applied StarDist [30], a commonly used cell segmentation method, to an example image collected from a human heart tissue sample that primarily consists of cardiomyocytes (Fig. 7a). The image was not processed by Vispro. To explore the heterogeneity of cell morphological features, we calculated various cell morphology metrics, such as area, perimeter, and elongation, based on segmented cell boundaries and projected these metrics into principal component analysis (PCA) space. Cells clearly separated into three distinct clusters, which does not align with the expected homogeneous morphology of cardiomyocytes. To investigate the cause, we visualized the locations of cells belonging to the three clusters and found that cells in two of the clusters were all mapped to fiducial marker regions. A zoomed-in view suggested that these segmentations were derived from fiducial markers and did not represent realistic cells (Fig. 7b). We then applied the same procedure to the image processed by Vispro. After processing, the fiducial markers were removed and the non-realistic cell segmentations were no longer present (Fig. 7b), resulting in homogeneous cell morphology features that better reflect the true nature of cardiomyocytes (Fig. 7c). These results suggest that Vispro can substantially improve the interpretation of cell morphology and the assessment of cellular heterogeneity.

We then applied StarDist to additional images with manually annotated tissue boundaries (Fig. 7d). StarDist identifies thousands of false positive cells when applied to the original images. In contrast, the number of false positive cells is significantly reduced when using images processed with Vispro for segmentation, highlighting the importance of removing unwanted technical artifacts from the images. Additional results of cell segmentation are provided in Additional file 2: Fig. S4.

### Vispro improves image registration

Image registration involves transforming a source image into the same coordinate system as a target image so that the two images are directly comparable. Many studies generate multiple ST samples from similar tissue types [31]. Registering these images to a common coordinate system enables researchers to compare spatial and imaging features across individuals. Similar to cell segmentation, image registration relies solely on imaging data, and its performance is influenced by the quality of the images.

**Fig. 8:**
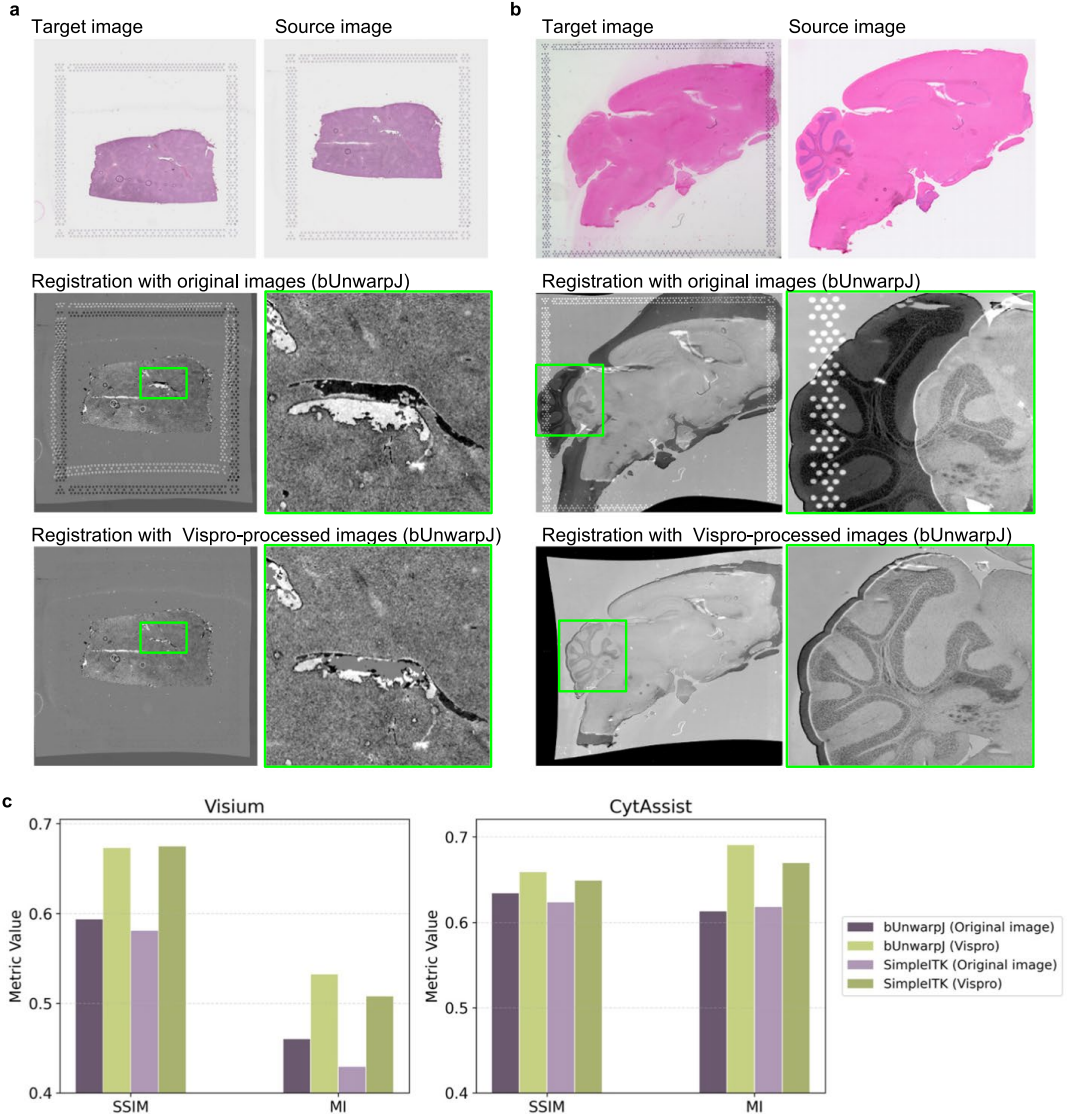
Image registration results. In **a** both the source and target images were generated by Visium. In **b** the source image was generated by Visium, while the target image was generated by the standard H&E workflow. The three rows in (**a** and **b**) display, from top to bottom: the original source images and target images, bUnwarpJ image registration results using the original images with zoomed-in views, and bUnwarpJ image registration results using Vispro-processed images with zoomed-in views. Image registration results display the overlay of the warped source image and the target image in grayscale. The source image is presented with inverted intensity values, while the target image is shown with normal intensity values. Differences between the two images are highlighted as regions of high contrast, appearing as intensely white or black areas. **c** SSIM and MI metrics evaluating registration performance using two registration algorithms, bUnwarpJ and SimpleITK

We tested the performance of two commonly used image registration methods, bUnwarpJ [32] and SimpleITK [33], using images processed by the full Vispro pipeline and the original images. We considered two image registration settings. In the first setting (Fig. 8a), both the source and target images were generated by the Visium workflow and included fiducial markers. In the second setting (Fig. 8b), the source image was generated by the Visium workflow and included fiducial markers, while the target image was generated by the standard H&E histology workflow and did not include fiducial markers. This second setting is particularly useful for Visium CytAssist [34], an instrument that facilitates the transfer of transcriptomic probes from standard glass slides to Visium slides. The performance of image registration was quantitatively evaluated using two numerical measures-structural similarity index (SSIM) and mutual information (MI) (Methods).

Figure 8 demonstrates the performance of image registration in the two settings. For each case, a small section of the registered image was zoomed in to highlight the differences in registration results. When fiducial markers are present, bUnwarpJ attempts to align both the fiducial markers and the tissue regions. Since the position and layout of fiducial markers are influenced by technical factors (e.g., tissue placement on the slide), this results in inferior registration of some tissue regions, as seen in the misaligned tissue structures within the zoomed-in areas. In contrast, the performance of image registration improves substantially with images processed by Vispro. By removing fiducial markers, the registration process focuses solely on the tissue regions, resulting in better alignment of the highlighted tissue structures. Along with improved SSIM and MI values for both bUnwarpJ and SimpleITK, these findings suggest that Vispro enhances the performance of image registration. Additional image registration results are provided in Additional file 2: Fig. S5.

**Fig. 9:**
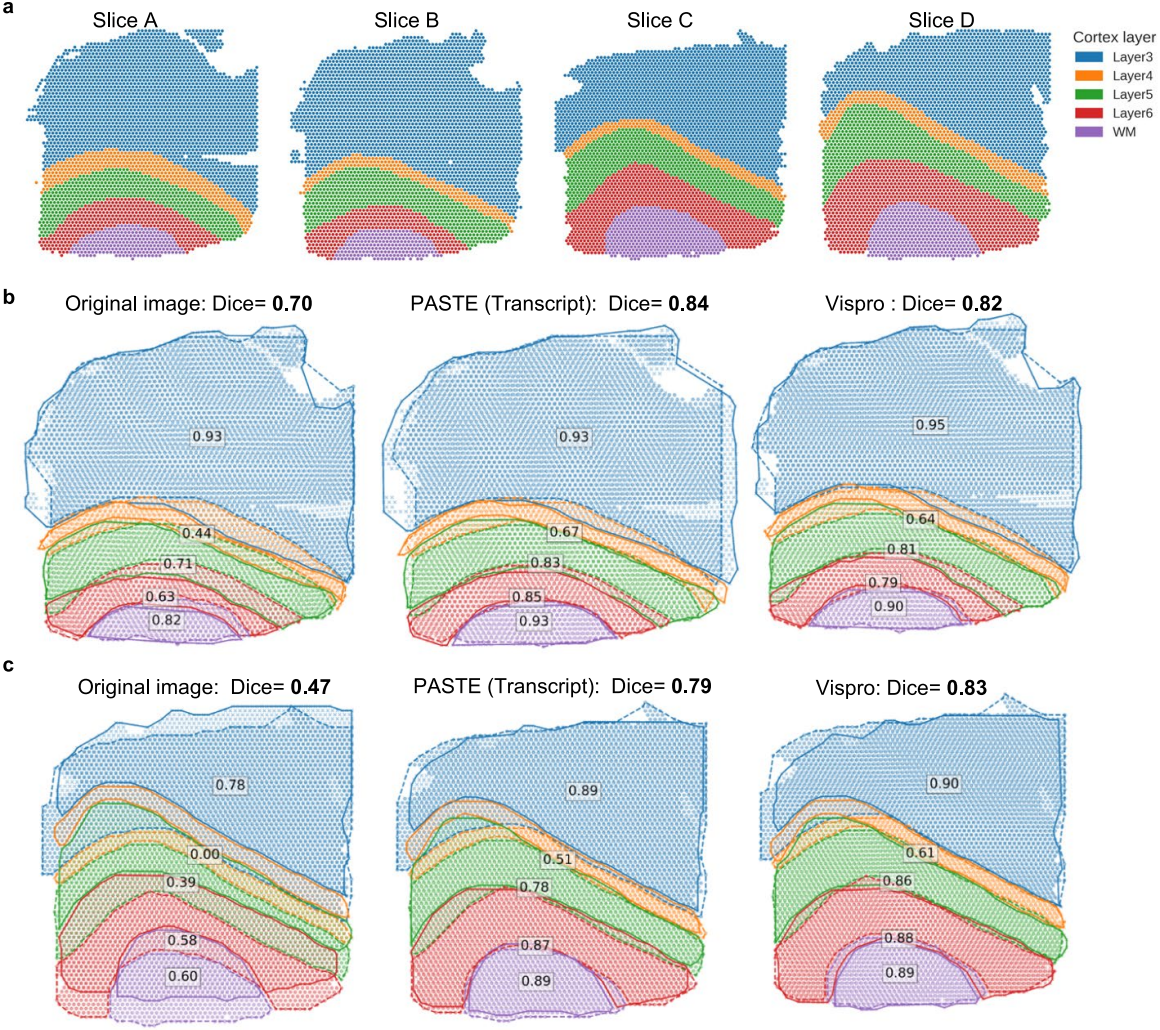
Evaluation of cortical layer agreement after image registration. **a** Manually annotated cortical layers in the four brain slices. **b**, **c** Registration results for the first pair of slices A and B (**b**) and for the second pair of slices C and D (**c**). From left to right, SimpleITK image registration using the original images, gene-based registration using PASTE, and SimpleITK image registration using Vispro-processed images. Dice coefficients are reported for each setting

To further assess how Vispro improves the biological interpretation of data after image registration, we collected ST datasets from two pairs of dorsolateral prefrontal cortex (DLPFC) slices along with their cortical layer annotations from a previous study [35] (Fig. 9a). We applied SimpleITK to perform image registration for each pair of slices using images processed by the full Vispro pipeline as well as the original images (Fig. 9b–c). We also included PASTE [36], a method for registering multiple ST datasets based on gene expression information in spatial spots. SimpleITK registration using Vispro-processed images is comparable to gene-based registration with PASTE and outperforms SimpleITK registration using the original images. Note that PASTE performs registration only for Visium spatial spots and cannot provide registration results for the whole image. In comparison, methods such as SimpleITK enable registration of whole images. By providing cleaner and better-aligned images, Vispro enhances the anatomical correspondence between slices, allowing more accurate integration of spatial gene expression data with tissue morphology.

### Vispro improves histology-based gene imputation

Finally, we demonstrate how Vispro enhances the results of TESLA [9], a method that imputes gene expression using histology image information. As examples, we examined the spatial expression patterns of HMGB1, a senescence-associated gene [37, 38], on a tissue slice from DLPFC. While the original Visium data provides only discrete, spot-level spatial expression patterns for these genes, TESLA generates a continuous spatial gene expression map across the entire tissue.

**Fig. 10:**
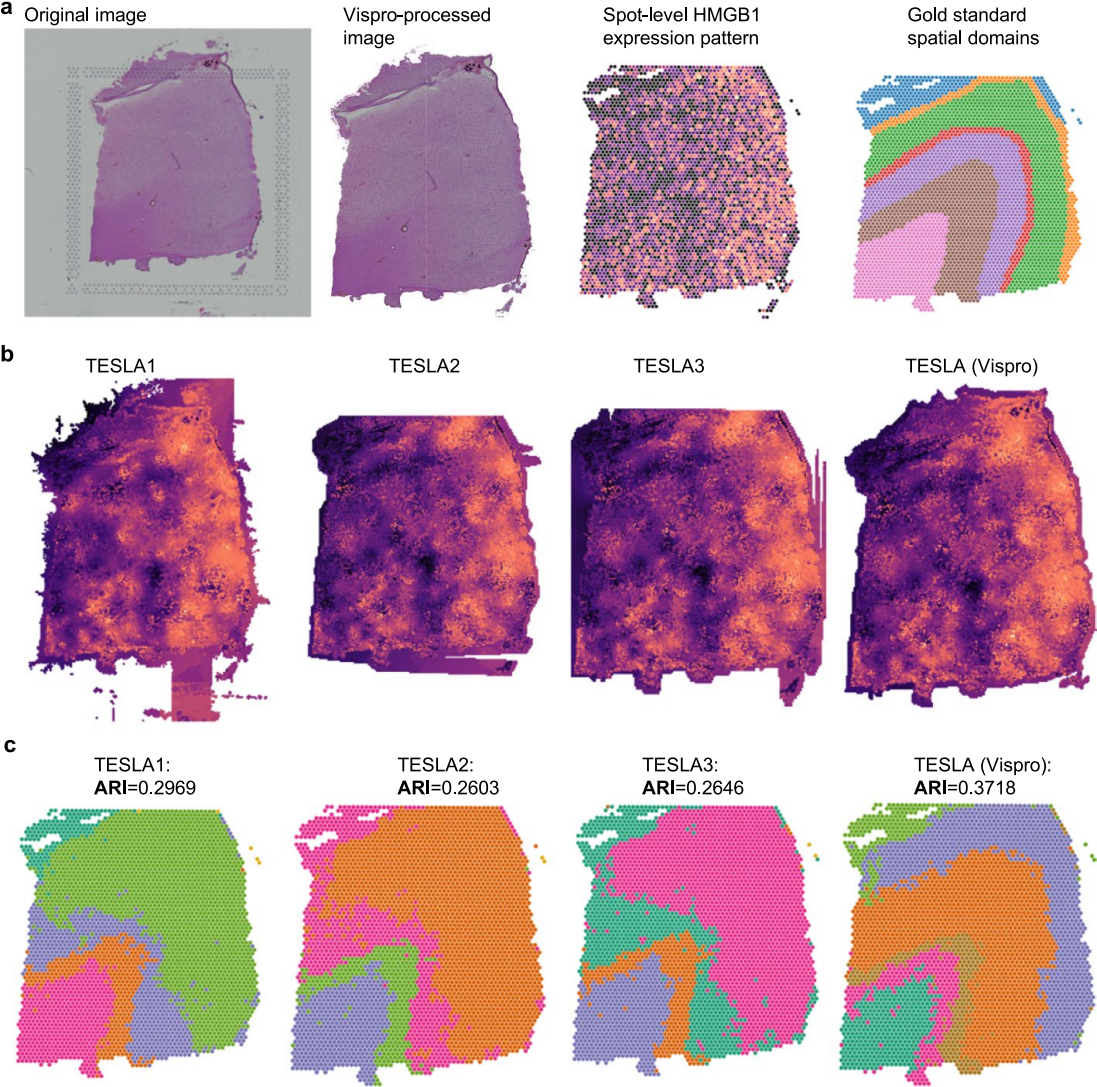
Image-based gene imputation results. **a** From left to right: the original image, Vispro-processed image, spot-level spatial gene expression patterns of HMGB1, and gold standard spatial domains. **b** From left to right: TESLA results using TESLA’s first tissue detection method (canny contour detection, TESLA1), TESLA results using TESLA’s second tissue detection method (scanning transcript contour by spot x, TESLA2), TESLA results using TESLA’s third tissue detection method (scanning transcript contour by spot y, TESLA3), and TESLA results using Vispro-processed images. **c** Detected spatial domains and ARI results. The order of the methods is the same as in (**b**)

We compared the performance of TESLA using images processed by different methods (Fig. 10a, b). TESLA offers three options for tissue detection prior to gene imputation; however, none of these approaches consistently produce reliable results. As a consequence, TESLA often imputes gene expression over broad regions that do not correspond to actual tissue, potentially leading to misleading interpretations of spatial gene expression patterns, especially in areas lacking real tissue. In contrast, when using Vispro-processed images, TESLA’s imputation is well-confined to true tissue regions, enabling more accurate and biologically meaningful interpretations of spatial gene expression. Additional results for gene imputation are provided in Additional file 2: Fig. S6.

We further evaluated how improved spatial gene expression patterns resulting from Vispro can enhance the interpretation of the data by performing spatial domain detection (Fig. 10c). We compared the computationally detected spatial domains using images processed by TESLA or Vispro with manually annotated cortical layers. Vispro-processed images result in spatial domains that are highly consistent with the gold standard, with an Adjusted Rand Index (ARI, Methods) of 0.37. In comparison, TESLA-processed images yield spatial domains that are less consistent with the gold standard and have a lower ARI. These results again demonstrate the importance of image preprocessing by Vispro, leading to better biological interpretation of the data.

### Vispro demonstrates generalizability and scalability

**Fig. 11:**
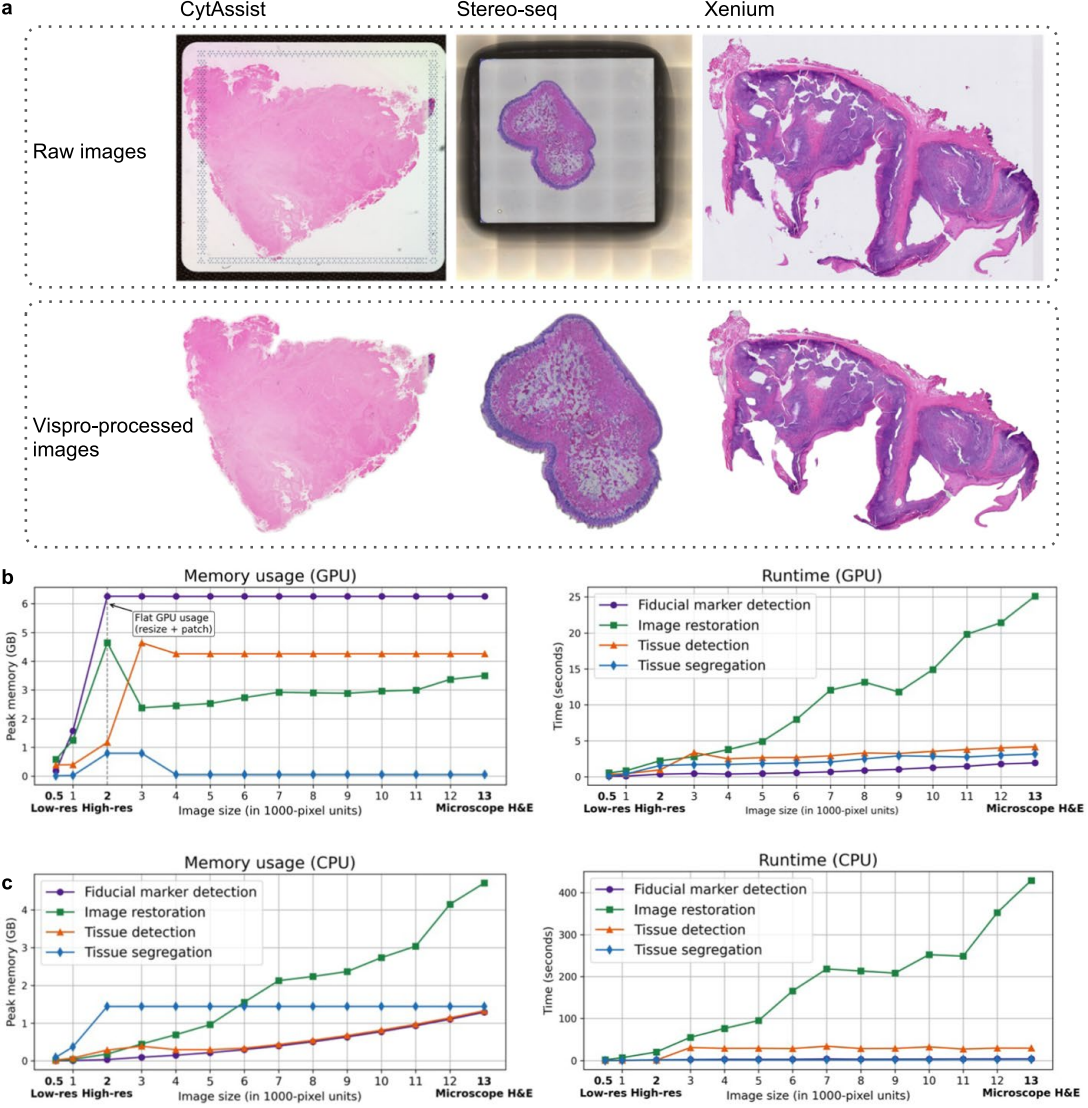
Generalizability and computational efficiency of Vispro across ST platforms and image sizes. **a** Representative raw input images from three platforms, 10× CytAssist, Stereo-seq, and 10× Xenium (top), and their corresponding Vispro-processed outputs (bottom). **b** Memory usage (left) and runtime (right) with GPU for four Vispro modules with increasing image sizes. **c** Memory usage (left) and runtime (right) with CPU for four Vispro modules with increasing image sizes

While Vispro is designed for images from 10× Visium, it can also be applied to images generated by other ST platforms, such as 10× CytAssist, Stereo-seq [39], and 10× Xenium [40]. Users can choose whether to perform fiducial marker detection and image restoration modules based on the presence of fiducial markers in the input image. Figure 11a presents representative images from ST platforms other than 10× Visium, shown both before and after processing with Vispro. Despite differences in resolution, staining intensity, and artifact distribution, Vispro consistently achieves reliable performance in removing background regions and recovering tissue regions.

To assess computational efficiency, we recorded the memory usage and runtime of individual Vispro modules on a GPU across a range of image sizes (Fig. 11b). To keep GPU memory usage within an 8 GB limit, images exceeding this threshold were resized for processing, and the image restoration step was performed using a patch-based approach. As a result, GPU memory usage across all four modules increases linearly at lower resolutions, then levels off for high-resolution images due to the patch-based implementation. Runtime remains under 5 s for most modules, except for image restoration, which may take up to 25 s for full-resolution, microscope-scale images. A similar analysis was performed using a CPU (Fig. 11c). Memory consumption remains relatively low, and while the runtime is notably longer than on GPU, it is still tractable. The image restoration module takes about 400 s even for the largest images.

## Discussion

While Vispro is designed to improve the quality of images in areas of fiducial markers and to detect tissue regions, it does not alter the images within spatial spots. This is because spatial spots are located on tissue regions and do not belong to fiducial markers, thus they are not affected by any Vispro processing module. As a result, Vispro does not impact the results of methods that only utilize images within spatial spots. For example, Additional file 2: Fig. S7 demonstrates spatial domain detection results from stLearn [41] and SiGra [13], two methods that use only imaging information from spatial spots. The detected spatial domains and the quantitative metric of ARI remain almost unchanged when Vispro-processed images are used instead of the original images. However, with the development of recent computational methods that integrate gene expression and imaging information, utilizing imaging information outside of spatial spots is becoming increasingly important. Vispro has the potential to substantially benefit these methods by providing high-quality whole-slide images.

## Conclusions

We present Vispro, an automated image processing tool for ST images generated by the 10× Visium platform. Vispro provides four consecutive modules to clean ST images and extract images of tissue regions. It accurately identifies fiducial markers and tissue regions in real examples, outperforming the default processing pipeline provided by 10×. We have demonstrated that processing images with Vispro can substantially improve the performance of downstream imaging analysis tasks, including cell segmentation, image registration, and gene imputation. Vispro achieves robust performance across diverse ST platforms. It is computationally efficient with or without GPU access, making it a practical preprocessing tool for large-scale biomedical imaging workflows.

Vispro represents the first comprehensive effort to develop an automated image preprocessing tool tailored for ST data. It systematically demonstrates how technical artifacts, such as fiducial markers and background noise, affect downstream analyses. To address these challenges, Vispro introduces a novel fiducial marker detection module powered by a customized U-Net trained on the first large-scale, diverse dataset of fiducial markers. Unlike existing approaches that are manual or limited in scope, Vispro integrates and optimizes multiple preprocessing steps into a scalable pipeline, achieving superior performance and practical utility in ST data analysis.

## Methods

### Vispro module 1: fiducial marker detection

The first step in Vispro involves detecting fiducial markers generated during the ST imaging process. Due to significant deformations in the shapes and positions of the markers, a deep learning approach was employed to capture their unique textures. We designed a neural network architecture based on the U-Net framework [20] to address the specific characteristics of markers across entire tissue slices. Significant effort was dedicated to assembling a robust training dataset to support model development. The original H&E image is extremely large, making it impractical for network training. Therefore, the model was trained on high-resolution sub-images to localize the marker areas, which were subsequently projected back to the original image scale.

#### Training data collection

We collected 167 datasets generated by the 10× Visium platform from 49 studies, which were downloaded from STOmics DB [42] and 10× Genomics website [43]. The training images included a total of 21 distinct tissue types, spanning both human and mouse origins as well as various biological conditions (Fig. 1f, g). The full list of studies is provided in Additional file 1: Table S2. To generate training annotations for fiducial marker areas, we applied the Hough circle transform algorithm [44], which uses pixel intensity to identify circular shapes in images. However, the algorithm produced numerous false positives in tissue regions and missed certain markers due to deformations or overlap with tissue areas. To address these limitations, we manually removed false positives and annotated markers missed by the Hough circle transform using the Labelme data annotation tool [45], resulting in a comprehensively annotated dataset containing 92,685 fiducial markers.

#### Neural network architecture

The neural network employed by Vispro is based on the U-Net architecture [20], a widely recognized neural network design for medical image segmentation. U-Net features a symmetric encoder-decoder structure optimized for efficient feature representation and reconstruction. The encoder progressively extracts hierarchical features through downsampling, while the decoder restores spatial resolution via upsampling, enabling the integration of fine-grained details with contextual information. This fully convolutional architecture ensures seamless feature extraction and reconstruction, making it well-suited for segmentation tasks.

The implementation of Vispro incorporates four UNetDown blocks, each designed to progressively reduce spatial resolution by half while increasing the number of feature channels from the initial 3 (RGB) to 32, 64, 128, and 256, respectively. Each block uses a $4\times 4$ convolution with a stride of 2, followed by instance normalization and ReLU activation [46], forming the core structure of the UNetDown block.

To address the specific challenges of fiducial marker segmentation, characterized by small-scale structures that are globally distributed but constrained to a square shape, we introduced targeted modifications to the network design. These adjustments prioritize retaining fine-grained details in the initial encoding layers while enhancing global shape awareness in the deepest encoding layers. The network structure is detailed below:

The input of the network is a 3-channel image, i.e., $I \in \mathbb {R}^{3 \times W \times H}$.

For the first encoding block, additional convolutional layers are added while avoiding dropout to preserve detailed feature extraction.

1 $$\begin{aligned} \text {Feature extraction:}~~~ & \dot{X}^1 = \text {Conv}_{3\times 3, s=1} \left( \text {Conv}_{4\times 4, s=2}(I) \right) , \nonumber \\ \text {Activation:}~~~ & \ddot{X}^1 = \text {ReLU}(\dot{X}^1). \end{aligned}$$

For the second and third encoding blocks ($i=2,3$), a foundation module is utilized.

2 $$\begin{aligned} \text {Feature extraction:}~~~ & \dot{X}^{i} = \text {Conv}_{4\times 4, s=2}(\ddot{X}^{i-1}), \nonumber \\ \text {Activation:}~~~ & \ddot{X}^{i} = \text {Dropout-ReLU-IN}(\dot{X}^{i}). \end{aligned}$$

For the fourth encoding block, additional convolutional layers are added to enhance feature extraction.

3 $$\begin{aligned} \text {Feature extraction:}~~~ & \dot{X}^4 = \text {Conv}_{3\times 3, s=1} \left( \text {Conv}_{4\times 4, s=2}(\ddot{X}^3) \right) , \nonumber \\ \text {Activation:}~~~ & \ddot{X}^4 = \text {Dropout-ReLU-IN}(\dot{X}^4). \end{aligned}$$

At the end of the encoding operations, additional bottleneck layers are incorporated at the deepest level of the network to further refine the receptive fields of the encoded features, as detailed below:

4 $$\begin{aligned} \text {Bottleneck layers:}~~~\dot{Y} = \text {Conv}_{3\times 3, s=1} \left( \text {Conv}_{3\times 3, s=1}(\ddot{X}^4) \right) . \end{aligned}$$

To reconstruct spatial context for fine-grained segmentation, the decoder progressively upsamples the bottleneck features through a series of four UNetUp blocks, mirroring the hierarchical structure of the encoder. At each scale, a $4 \times 4$ deconvolution operation is applied to double the spatial resolution of the learned features while sequentially reducing the feature channels from 256 to 128, 64, 32, and 16. These operations are followed by activation functions and skip connections, which seamlessly integrate high-resolution features from the encoder. This approach ensures the preservation of spatial details and enriches the reconstruction process with complementary contextual information.

Formally, the operations at the first decoding block are defined as follows, with additional convolutional layers applied to both the decoding features and the skip features to enhance global attention:

5 $$\begin{aligned} \text {Feature decoding:}~ & \dot{Z}^1 = \text {Conv}_{3\times 3}\left( \text {DeConv}_{4\times 4}(\dot{Y})\right) , \nonumber \\ \text {Activation:}~ & \ddot{Z}^1 = \text {Dropout-ReLU-IN}(\dot{Z}^1), \nonumber \\ \text {Skip connection:}~ & \ddddot{Z}^1 = \ddot{Z}^1 + \text {Conv}_{1\times 1}(\ddot{X}^3). \end{aligned}$$

For the second decoding block:

6 $$\begin{aligned} \text {Feature decoding:}~ & \dot{Z}^2 = \text {DeConv}_{4\times 4}(\ddddot{Z}^1), \nonumber \\ \text {Activation:}~ & \ddot{Z}^2 = \text {Dropout-ReLU-IN}(\dot{Z}^2), \nonumber \\ \text {Skip connection:}~ & \dddot{Z}^2 = \ddot{Z}^2 + \ddot{X}^2. \end{aligned}$$

For the third decoding block:

7 $$\begin{aligned} \text {Feature decoding:}~ & \dot{Z}^3 = \text {DeConv}_{4\times 4}(\dddot{Z}^2), \nonumber \\ \text {Activation:}~ & \ddot{Z}^3 = \text {ReLU-IN}(\dot{Z}^3), \nonumber \\ \text {Skip connection:}~ & \dddot{Z}^3 = \ddot{Z}^3 + \ddot{X}^1. \end{aligned}$$

For the fourth decoding block:

8 $$\begin{aligned} \text {Feature decoding:}~ & \dot{Z}^4 = \text {Conv}_{3\times 3}\left( \text {DeConv}_{4\times 4}(\dddot{Z}^3)\right) , \nonumber \\ \text {Activation:}~ & \ddot{Z}^4 = \text {ReLU-IN}(\dot{Z}^4), \nonumber \\ \text {Skip connection:}~ & \dddot{Z}^4 = \ddot{Z}^4 + I. \end{aligned}$$

Each decoder layer processes the learned feature maps $\dot{Z}^i$ while incorporating feature maps $\dot{X}^i$ from the corresponding encoder layers via skip connections. These skip connections retain spatial details that are otherwise lost during downsampling, enabling the network to effectively merge high-resolution spatial features with deeper contextual information. This synergy significantly enhances the network’s capacity to reconstruct fine-grained features with precision, a critical advantage for segmentation tasks. The Vispro architecture further ensures that the network adeptly captures both intricate details and global context, making it particularly well-suited for segmenting small fiducial markers while maintaining their global shape constraints.

#### Neural network output

The output of the decoder is a 19-channel feature map at full resolution, comprising 16 learned feature channels and 3 channels from the original input image, i.e., $\dddot{Z}^4 \in \mathbb {R}^{19 \times W \times H}$. This feature map is passed through a learnable final layer, which generates the marker segmentation mask $\hat{M}$:

9 $$\begin{aligned} \hat{M} = \text {sigmoid}\left( \text {Conv}_{4 \times 4, s=1}(\dddot{Z}^4)\right) \in [0, 1]^{W \times H} \end{aligned}$$

#### Loss function

We employed a combined loss function, consisting of Dice Loss [47] and Focal Loss [48], to evaluate the predicted marker mask, $\hat{M}$, against the gold standard mask, *M*, obtained through manual annotation. The gold standard mask, *M*, is a binary mask where 1 represents the fiducial marker region and 0 represents the background region. In contrast, $\hat{M}$ contains values in the range (0, 1), where values closer to 1 indicate a high likelihood of the marker region, and values closer 0 indicate a high likelihood of the background region.

The Dice Loss, widely used in segmentation tasks, evaluates the overlap between the target regions in the two masks. To calculate the Dice Loss, the predicted mask, $\hat{M}$, is first binarized by applying a threshold of 0.5, resulting in a binary prediction mask, $\hat{M}_b$. The Dice Loss is then computed as follows:

10 $$\begin{aligned} \text {Dice Loss:} \quad L_d = 1 - \frac{2 \cdot |\hat{M}_b \cap M| + \epsilon }{|\hat{M}_b| + |M| + \epsilon } \end{aligned}$$

where $\epsilon$ is a smoothing factor set to $\epsilon = 1.0$ to stabilize the loss value, especially for small or empty masks.

For the Focal Loss, it builds upon the pixel-wise Binary Cross-Entropy (BCE) Loss and introduces additional parameters to emphasize hard-to-classify markers while down-weighting easy-to-classify markers by adjusting the loss contribution of each example. Specifically, the BCE Loss is defined as:

11 $$\begin{aligned} \text {BCE Loss:} \quad L_{\text {BCE}} = - \frac{1}{N} \sum \limits _{i=1}^{N} \left[ y_i \cdot \log (\hat{y}_i) + (1 - y_i) \cdot \log (1 - \hat{y}_i) \right] , \quad y_i \in M, \, \hat{y}_i \in \hat{M} \end{aligned}$$

where $N$ is the total number of pixels, $y_i$ represents the ground truth label for pixel $i$, and $\hat{y}_i$ is the corresponding predicted value from the neural network.

Focal Loss introduces weighting terms that dynamically adjust the loss contributions based on the predicted values of the network. This adjustment is achieved through the following formulation:

12 $$\begin{aligned} \text {Focal Loss:} \quad L_f = - \frac{1}{N} \sum \limits _{i=1}^{N} \left[ \alpha \cdot y_i \cdot (1 - \hat{y}_i)^\gamma \cdot \log (\hat{y}_i) + (1 - \alpha ) \cdot (1 - y_i) \cdot \hat{y}_i^\gamma \cdot \log (1 - \hat{y}_i) \right] \end{aligned}$$

where $\gamma$ controls the level of focus on hard examples, and $\alpha$ is a class-level weighting factor used to prioritize the target class. In Vispro, we set $\alpha = 0.95$ and $\gamma = 3$.

By inversely scaling the weights based on the confidence levels of predictions, the loss function reduces the impact of well-classified samples while emphasizing harder and misclassified examples. This adaptive weighting mechanism ensures that the network prioritizes challenging samples during training, effectively balancing the contributions of both easy and hard examples to the overall loss.

The total loss used to train the network combines the Dice Loss and Focal Loss, defined as follows:

13 $$\begin{aligned} \text {Total Loss:} \quad L_{\text {total}} =\lambda \cdot L_d + (1 - \lambda ) \cdot L_f \end{aligned}$$

where $\lambda$ is a weighting parameter that balances the contributions of the two loss components, set to $\lambda = 0.9$ in Vispro.

#### Training procedure

Since markers located in the background region are more numerous and easier to classify, whereas markers overlapping with tissue regions are fewer and harder to distinguish, we implemented a sampling layer to increase the frequency of challenging samples during network training. Specifically, we computed a slice-wide marker overlap factor, defined as the ratio of in-tissue markers to the total number of markers. Based on this factor, we categorized images into three difficulty levels: hard examples (marker factor > 0.8), moderate examples (0.3 < marker factor $\le$ 0.8), and easy examples (marker factor < 0.3). To ensure the model effectively learns from the most challenging cases, we increased the occurrence of hard samples threefold and moderate samples twofold in the training dataset.

During training, all images underwent carefully designed data augmentation strategies to enhance data variability and improve model robustness. These augmentations included random flipping (both left-right and up-down), scaling (0.8 to 1.0 times the original size), rotation ($-10^\circ \text { to } +10^\circ$), brightness adjustment (0.5 to 1.2 times the original value), and Gaussian blurring (with a standard deviation randomly selected between 0 and 1). The model is trained using the Adam optimizer [49] with a learning rate of $10^{-4}$. The optimizer’s parameters include $\beta _1 = 0.5$ and $\beta _2 = 0.999$, which control the exponential decay rates of the first- and second-order moment estimates of the gradients, respectively. These settings ensure stable convergence during training. The network was trained for 800 epochs.

### Vispro module 2: image restoration

The second step in Vispro involves performing image restoration on the marker-covered regions of the images. For this task, we leverage LaMa, a deep learning model specifically designed for image inpainting [50]. Image inpainting is a computer vision technique that aims to fill in missing or corrupted regions of an image. The fundamental principle involves learning the surrounding textures and global image semantics to generate visually plausible content for the missing regions.

In this module, we incorporate the pre-trained model and parameters of LaMa [51], which employs Fourier Convolutions [52] to address the limitations of conventional inpainting methods that often struggle to generalize across varying resolutions. This approach ensures consistent performance on both low- and high-resolution images, enabling pre-training on large-scale natural image datasets while seamlessly adapting to ST images, which are typically high resolution.

The input to the model consists of the original image *I* and the predicted binary mask $\hat{M}$ generated by Vispro Module 1. The output is an RGB image $\hat{I}$, where the fiducial marker regions are filled with plausible textures inferred from the surrounding image content.

### Vispro module 3: tissue detection

For the task of tissue detection, we employed the deep learning tool backgroundremover [53], which is based on the U²-Net model [21], a state-of-the-art salient object detection framework. The incorporation of Residual U-blocks (RSU) [21] enhances the network’s capacity for efficient hierarchical feature learning, ensuring precise representation of fine-grained details alongside broader spatial patterns, making it particularly adept at robust tissue segmentation, especially in high-resolution images where traditional models frequently struggle to achieve a balance between detail preservation and global contextual accuracy.

By default, Vispro uses the unet2 model and resizes images to 320 $\times$ 320 to optimize computational efficiency. For datasets with small tissue regions (less than 100 $\times$ 100 pixels), Vispro increases the image size to 1000 $\times$ 1000 and switches to the u2netp model. This adjustment enhances detection accuracy for finer structures and thinner tissue regions.

The input to the model is the restored RGB image $\hat{I}$ produced by Vispro Module 2, and the outputs are a tissue mask $\hat{M}_t$ with values in the range [0, 1] and a blended RGBA image $\hat{I}_t$ that visually highlights the detected tissue regions.

### Vispro module 4: disconnected tissue segregation

We identified disconnected components in the tissue probability mask $\hat{M}_t$ using the OpenCV library. Specifically, the probability mask was binarized using a tissue threshold value (default set to 0.8). The label function was then employed to count and label the disconnected regions in the binary mask. This function performs a brute-force search to determine pixel connectivity, iterating through each pixel in the mask and checking for 8-connectivity (including horizontal, vertical, and diagonal neighbors) to ascertain whether adjacent pixels belong to the same component. Once a connected group of pixels is identified, the algorithm assigns a unique integer label to the corresponding component. The output is a labeled mask in which each connected component is represented by a distinct integer value.

The input to this module is the tissue probability mask $\hat{M}_t$ produced by Vispro Module 3, and the output is a refined segmentation mask $\hat{M}_s$, represented with integer values.

To improve the quality of the labeled mask, an additional step was introduced to remove small components, often artifacts caused by staining errors or computational inaccuracies near the tissue boundary. These small components were filtered out using a preset area threshold (default set to 20). Any component with an area below this threshold was excluded from the labeled mask. This refinement step ensures that the labeled mask more accurately represents tissue regions and minimizes noise.

### Competing methods

#### 10× standard processing pipeline

In the 10× pipeline, fiducial markers are processed using Space Ranger [15] and Loupe Browser [16], which generate a layered image. This image includes a fiducial frame template with red circles overlaid on the original H&E image to highlight fiducial marker areas. These tools can automatically detect and highlight the fiducial regions, providing a visual reference for their locations on the tissue. However, they usually require complex model inputs, including gene count data, slide version information, and images. Additionally, in cases where fiducial markers are obstructed or tissue boundaries are unclear, manual alignment is often necessary.

To integrate 10×’s aligenment result into our workflow for evaluation, we first perform an RGB thresholding operation on the layered image to isolate the red color component representing the fiducial markers. This step extracts the red circles by identifying pixels that fall within a predefined range of RGB color intensities (R >180, G<30, and B<30). Once the red components are isolated, we apply a circle detection of Hough circle transform to detect the circular fiducial markers. This algorithm scans the image for circular shapes by identifying edge pixels and fitting circles to the detected contours. After detecting the circles, we fill in each circle area to create a binary mask. In this mask, pixel values are set to 1 within the fiducial marker regions and 0 elsewhere, mimicking the format used in our pipeline. This binary mask is then used for further evaluation, enabling a direct comparison of the performance between the 10× pipeline and Vispro.

#### Hough circle transform

The Hough circle transform [44] is a classical technique for detecting circular patterns in images by mapping edge-detected points into a parameter space. It identifies curves through a voting process in a discretized accumulator space, making it well suited for detecting parametric shapes such as circles. We adopted the OpenCV implementation with default settings to detect circular features like fiducial markers. This method serves as a non-learning-based baseline in our comparative evaluation.

#### Cellpose

Cellpose [23] is a generalist algorithm for cell segmentation that performs well across a broad range of cell types and imaging modalities. It models spatial gradients (vector flows) from cell interiors to boundaries to guide mask generation, enabling generalizable performance across domains. We used the pre-trained Cellpose model with the diameter parameter fixed at 25, which corresponds closely to the average fiducial marker size in our dataset. This standard configuration ensures a consistent and fair evaluation.

#### CircleNet

CircleNet [24] is a deep learning-based framework specifically designed for circle detection, leveraging the hourglass network architecture to capture multi-scale spatial features. The model predicts circle centers and radii directly from the image using convolutional outputs. In our study, we used the hourglass architecture with the pre-trained model provided from the MoNuSeg dataset. This setup enables robust detection of circular structures and serves as a learning-based benchmark.

#### Baseline U-Net

For the baseline U-Net, we follow the design of pix2pix [46], a widely used U-Net variant for image processing tasks. We adopt its U-Net generator as the baseline for marker segmentation and compare its results with the tailored architecture in Vispro. The network generates fiducial marker masks similar to Vispro, enabling a direct comparison of performance.

#### Deep image prior

Deep Image Prior [26] (DIP) leverages the structure of a convolutional neural network to perform image restoration without requiring any pre-training. The method is based on the insight that the network architecture itself captures natural image statistics, enabling it to denoise or inpaint images without external datasets. We followed the recommended protocol and trained DIP for 2000 iterations per image to inpaint corrupted or missing regions. This approach serves as an unsupervised benchmark for restoration performance.

#### Stable diffusion model

Stable Diffusion [27] is a generative model capable of high-fidelity semantic inpainting. It operates by learning a latent representation of images through denoising score matching and iteratively generating plausible content conditioned on a text prompt. We employed the inpainting-specific model runwayml/stable-diffusion-inpainting with the prompt “Fill the masked region seamlessly.” This approach enables content-aware filling of masked tissue regions and serves as a generative baseline.

#### Otsu

Otsu [17] is a widely used histogram-based thresholding algorithm for binarizing grayscale images. It determines the optimal threshold by maximizing the inter-class variance between foreground and background pixels, making it effective for images with bimodal histograms. We applied OpenCV’s implementation to provide a fast, unsupervised baseline for separating tissue from background.

#### SAM

The Segment Anything Model [18] (SAM) is a prompt-driven segmentation framework based on vision transformers. It generates segmentation masks by attending to user-provided prompts, such as points or bounding boxes, using a pre-trained image encoder to generalize across diverse object types. We evaluated SAM in two settings: tissue segmentation and tissue segregation. For tissue segmentation, a binary task, we used the image center as a point prompt to extract a confident primary region, which was treated as the tissue area. For tissue segregation, a multi-class segmentation task, we applied the model’s default “segment anything” mode across the entire image. Both settings used the *sam_vit_h_4b8939.pth* model.

#### TESLA

Although TESLA [54] was originally developed for gene expression imputation in spatial transcriptomics, it includes a built-in tissue detection module that combines image-derived and transcriptome-derived strategies for boundary estimation. This module incorporates three techniques: (1) Canny edge detection via OpenCV, (2) horizontal projection of transcript spot density along the *x*-axis, and (3) vertical projection along the *y*-axis. We applied all three techniques using the default settings.

### Evaluations

#### Fiducial marker identification

To evaluate the accuracy of fiducial marker segmentation, we computed the pixel-level Intersection over Union (IoU) using the manually annotated gold standard as a reference. The IoU is a metric that measures the overlap between the predicted mask and the ground truth mask at the pixel level. It is defined as the ratio of the number of correctly predicted pixels (i.e., the intersection of the predicted and ground truth masks) to the total number of pixels in the union of the predicted and ground truth masks:

14 $$\begin{aligned} \text {IoU} = \frac{\sum _{i,j} \left( P_{i,j} \wedge G_{i,j} \right) }{\sum _{i,j} \left( P_{i,j} \vee G_{i,j} \right) } \end{aligned}$$

where $P_{i,j}$ represents the predicted mask at pixel location $(i, j)$, $G_{i,j}$ represents the ground truth mask at pixel location $(i, j)$, $\wedge$ denotes the logical AND operation (i.e., the number of pixels where both $P_{i,j} = 1$ and $G_{i,j} = 1$, representing the intersection), and $\vee$ denotes the logical OR operation (i.e., the number of pixels where either $P_{i,j} = 1$ or $G_{i,j} = 1$, representing the union). A higher IoU value indicates a higher segmentation accuracy, as it shows that the predicted mask aligns more closely with the ground truth mask.

All images were divided into 12 groups for 12-fold cross-validation. In each iteration, images from 11 groups were used for training, while images from the remaining group were used for testing.

#### Identifying tissue regions

To assess the accuracy of tissue region segmentation, we manually annotated tissue regions on 21 tissue slices (Additional file 1: Table S3), encompassing a spectrum of difficulty from straightforward to challenging cases.

To quantitatively assess segmentation performance, we employed three metrics that capture both regional accuracy and boundary shape characteristics: Intersection over Union (IoU), Hausdorff Distance (HD), and Perimeter Ratio (PerimRatio).

IoU measures the overlap between the predicted mask *P* and the ground truth mask *G*, defined as:

15 $$\begin{aligned} \textrm{IoU}(P, G) = \frac{|P \cap G|}{|P \cup G|}. \end{aligned}$$

The average IoU is computed by taking the mean of $\textrm{IoU}(P_i, G_i)$ over all samples.

Hausdorff Distance measures the maximum distance between points on the predicted boundary $\partial P$ and the closest points on the ground truth boundary $\partial G$:

16 $$\begin{aligned} \textrm{HD}(P, G) = \max \left\{ \sup _{p \in \partial P} \inf _{g \in \partial G} d(p, g),\ \sup _{g \in \partial G} \inf _{p \in \partial P} d(g, p) \right\} , \end{aligned}$$

where $d(\cdot ,\cdot )$ is the Euclidean distance. The average HD is obtained across all samples.

To assess boundary complexity relative to the ground truth, the perimeter ratio is defined as:

17 $$\begin{aligned} \textrm{PerimRatio}(P, G) = \frac{|\partial P|}{|\partial G|}. \end{aligned}$$

This ratio identifies cases of over- or under-segmentation with excessive or insufficient boundary delineation.

#### Segregating disconnected tissue regions

To evaluate the accuracy of tissue segregation, we computed both the IoU and the difference in the number of connected components between the predicted mask *P* and the ground truth *G*. The IoU is defined above. The difference in the number of components is calculated as:

18 $$\begin{aligned} \textrm{CompDiff}(P, G) = \left| N(P) - N(G) \right| , \end{aligned}$$

where $N(\cdot )$ denotes the number of connected components in a binary mask. This metric captures both over-segmentation (excess components) and under-segmentation (merged regions), providing a measure of the topological fidelity of the prediction.

#### Cell segmentation

To evaluate cell segmentation performance, we utilized StarDist [30, 55, 56] to detect cells in both the original H&E images and the images processed by Vispro. Evaluation was performed on 20 tissue slices with microscopy-resolution images (Additional file 1: Table S3). StarDist is a state-of-the-art segmentation algorithm designed for star-convex object detection in both 2D and 3D images, making it particularly well-suited for accurately segmenting objects with varying shapes and sizes, such as nuclei in histopathological images. We employed the 2D_versatile_he model, pre-trained for H&E-stained histological images. The main model parameters were configured as follows: block_size was set to 4096, prob_threshold to 0.01, and nms_threshold to 0.001.

To compute the PCA projections of cell morphological features, we extracted the following descriptors: area, perimeter, elongation, eccentricity, circularity, solidity, extent, aspect ratio, and compactness. Each feature was then standardized to have a mean of 0 and a standard deviation of 1 across cells. PCA was applied to the scaled feature set, and the projections onto the first two principal components were visualized.

For cell segmentation results obtained by each method, we calculated the number of segmented cells falling outside of the manually annotated tissue regions.

#### Image registration

We evaluated the accuracy of image registration in both same-modal and cross-modal settings. For the same-modal setting, we used 20 pairs of Visium images from our collected dataset (Additional file 1: Table S3), where each pair originated from the same study and shared similar textures. For the cross-modal setting, we collected 13 pairs of images from the Visium CytAssist dataset (Additional file 1: Table S4). This dataset involves overlaying images of standard histological glass slides with those processed into Visium slides. While structurally identical, the pairs exhibit slight texture differences, making them ideal for testing cross-modal registration performance.

We included two competing methods for evaluation. The first is bUnwarpJ registration [57], available within the ImageJ/Fiji software [32], for image registration. bUnwarpJ performs 2D image registration using elastic deformations modeled by B-splines, ensuring invertibility through a consistency constraint. We used the stable version (2.6.13) of the software, configuring the initial deformation to coarse, the final deformation to super fine, while leaving all other parameters at their default settings. The second is SimpleITK, a tool for image registration that supports rigid, affine, and deformable transformations with customizable similarity metrics, optimizers, and interpolators. We used version 2.4.0 of the software with default settings.

Three metrics were used for quantitatively evaluations. The first metric is the Structural Similarity Index (SSIM), which measures the similarity between two images by comparing local patterns of pixel intensities, normalized for differences in luminance and contrast. SSIM is particularly sensitive to pixel misalignment, making it a robust metric for evaluating registration performance, especially in the context of same-modal image registration. For the multi-channel data in our case (i.e., RGB with three channels), SSIM is calculated separately for each channel, and the final metric is obtained by averaging the scores across all channels. This approach ensures a comprehensive evaluation of structural similarity for color images. For each channel, the SSIM is mathematically defined as:

19 $$\begin{aligned} \text {SSIM}(I_x, I_y) = \frac{1}{N} \sum \limits _{k=1}^{N} \frac{(2\mu _x^k \mu _y^k + c_1)(2\sigma _{xy}^k + c_2)}{(\mu _x^{k^2} + \mu _y^{k^2} + c_1)(\sigma _x^{k^2} + \sigma _y^{k^2} + c_2)}, \end{aligned}$$

where $I_x$ and $I_y$ are the two input images, *N* is the total number of local image patches. $\mu _x^k$ and $\mu _y^k$ represent the local means, $\sigma _x^k$ and $\sigma _y^k$ are the local standard deviations, and $\sigma _{xy}^k$ denotes the cross-covariance of the intensity values in the *k*th patch of $I_x$ and $I_y$. The constants $c_1$ and $c_2$ are stability parameters introduced to prevent division by zero.

The second metric is Mutual Information (MI), which quantifies the amount of information shared between two random variables and is widely used in image registration tasks to measure the similarity between images. As MI is calculated from whole-image pixel patterns, it excels at identifying alignment by leveraging structural and contextual consistency rather than relying on simple intensity correspondence, making it particularly effective for estimating registration tasks involving images from different modalities. To handle the continuous intensity values in the image pair $I_x$ and $I_y$, the pixel intensities are first discretized into bins using a 2D histogram. Each bin represents a range of intensity values, and the histogram captures the co-occurrence of intensity values from the two images at corresponding spatial locations. The 2D histogram is then normalized to estimate the joint probability distribution *p*(*x*, *y*), as well as the marginal probabilities *p*(*x*) and *p*(*y*), where *x* and *y* represent the bins of intensity values from $I_x$ and $I_y$, respectively. These probabilities are then used to compute MI.

Formally, MI is calculated as follows:

20 $$\begin{aligned} \text {MI}(I_x, I_y) = \sum \limits _{x \in I_x} \sum \limits _{y \in I_y} p(x, y) \log \left( \frac{p(x, y)}{p(x) p(y)} \right) , \end{aligned}$$

The third metric is the Dice score, which is used to assess the agreement between expert-annotated cortical layers after performing image registration on the DLPFC dataset [35]. The Dice score is a widely used metric for measuring the similarity between two sets and is particularly well-suited for assessing multi-class area overlap in spatial segmentation tasks. Specifically, we extracted the convex hulls of manually annotated cortical layers in both the target and source images after performing image registration with different methods. These convex hulls were converted into image-format masks, where distinct pixel values represented different tissue layers. The Dice score was then computed to quantify the spatial overlap between corresponding layers.

Given two segmentation masks, $I_x$ and $I_y$, corresponding to the target image and the source image after registration, respectively. The Dice score is defined as:

21 $$\begin{aligned} \textrm{Dice}(I_x, I_y) = \frac{1}{C} \sum \limits _{c=1}^{C} \frac{2 \cdot |(I_x = c) \cap (I_y = c)|}{|(I_x = c)| + |(I_y = c)|}. \end{aligned}$$

where $(I_x = c)$ and $(I_y = c)$ denote the sets of pixels assigned to class $c$ in the target and source images, respectively. This formulation captures both over-segmentation and under-segmentation across tissue regions and reflects the topological fidelity of the registration.

#### Gene imputation

We employed the TESLA software for gene imputation [54]. TESLA leverages the Canny edge detection algorithm from the OpenCV library for contour detection and also offers alternative methods based on the spatial locations of transcripts. We tested all three contour detection methods provided by TESLA and conducted a detailed visual comparison of the imputation outcomes (Additional file 1: Table S5). For consistency, the imputation resolution was set to 50 during implementation.

After performing gene imputation using TESLA, we further conducted spatial domain detection. Specifically, we utilized the standard Seurat pipeline [58] to perform library size normalization, log transformation, highly variable gene selection, and PCA on the imputed gene expression counts. We then applied *k*-means clustering to the PCA embeddings to obtain cell cluster labels. The resulting spatial domain labels were then projected back onto the original spot coordinates based on nearest-neighbor matching. Finally, the Adjusted Rand Index (ARI) was used to compare the predicted spatial domains with manual annotations.

The ARI is defined as:

22 $$\begin{aligned} \textrm{ARI} = \frac{\sum _{ij} \left( {\begin{array}{c}n_{ij}\\ 2\end{array}}\right) - \left[ \sum _i \left( {\begin{array}{c}a_i\\ 2\end{array}}\right) \sum _j \left( {\begin{array}{c}b_j\\ 2\end{array}}\right) / \left( {\begin{array}{c}n\\ 2\end{array}}\right) \right] }{\frac{1}{2} \left[ \sum _i \left( {\begin{array}{c}a_i\\ 2\end{array}}\right) + \sum _j \left( {\begin{array}{c}b_j\\ 2\end{array}}\right) \right] - \left[ \sum _i \left( {\begin{array}{c}a_i\\ 2\end{array}}\right) \sum _j \left( {\begin{array}{c}b_j\\ 2\end{array}}\right) / \left( {\begin{array}{c}n\\ 2\end{array}}\right) \right] }, \end{aligned}$$

where $n_{ij}$ is the number of elements shared between predicted cluster $i$ and true cluster $j$, $a_i$, and $b_j$ are the numbers of elements in predicted cluster $i$ and true cluster $j$, respectively, and $n$ is the total number of elements. The ARI ranges from 0 (random labeling) to 1 (perfect match), providing a robust measure of clustering agreement.

## Supplementary information

Additional file 1: Table S1. List of spatial transcriptomics analysis tools. Provides an overview of existing tools, including name, publication year, citation, and usage of histology images. Also indicates tool capabilities across six functions: image processing, full tissue extent handling, fiducial marker detection, image restoration, tissue detection, and disconnected tissue segregation. Table S2. List of Visium data sources. Lists the Visium datasets used for training Vispro, including dataset names, sources, species, tissue types, tissue conditions, number of images, and unique sample IDs. Table S3. Evaluation information. Lists the sample IDs of Visium datasets used for evaluating tissue area identification, cell segmentation, and image registration modules. Table S4. List of CytAssist data sources. Lists the CytAssist datasets used to evaluate the image registration module and demonstrate generalizability, including dataset names, sources, species, tissue types, and number of images. Table S5. List of gene imputation data sources. Includes dataset names, sources, species, tissue types, and number of images used for gene imputation task. Table S6. List of Stereo-seq data sources. Includes dataset sources, species, tissue types, and number of images from Stereo-seq data used to demonstrate generalizability. Table S7. List of Xenium data sources. Includes dataset sources, species, tissue types, and number of images from Xenium data used to demonstrate generalizability.

Additional file 2: Figure S1. Additional image restoration results. Figure S2. Additional tissue detection results. Figure S3. Additional disconnected tissue segregation results. Figure S4. Additional cell segmentation results. Figure S5. Additional image registration results. Figure S6. Additional image-based gene imputation results. Figure S7. Spatial domain detection using SiGra and stLearn on original and Vispro-processed images.

## Data Availability

Vispro can be freely accessed at Github [59] and archived at Zenodo [60] under the MIT license. The Github repository includes detailed instructions for usage and any required dependencies. The datasets used in this study are listed in Additional file 1: Table S2.
