# Supplementary material for: Vispro improves imaging analysis for Visium spatial transcriptomics
Source: Genome Biol. 2025 Jun 18;26:173. doi: 10.1186/s13059-025-03648-w (PMC12177973; doi:10.1186/s13059-025-03648-w)
Supplement: Supplementary file 2 — Additional file 2: Figure S1. Additional image restoration results. Figure S2. Additional tissue detection results. Figure S3. Additional disconnected tissue segregation results. Figure S4. Additional cell segmentation results. Figure S5. Additional image registration results. Figure S6. Additional image-based gene imputation results. Figure S7. Spatial domain detection using SiGra and stLearn on original and Vispro-processed images. [file 13059_2025_3648_MOESM2_ESM.pdf]

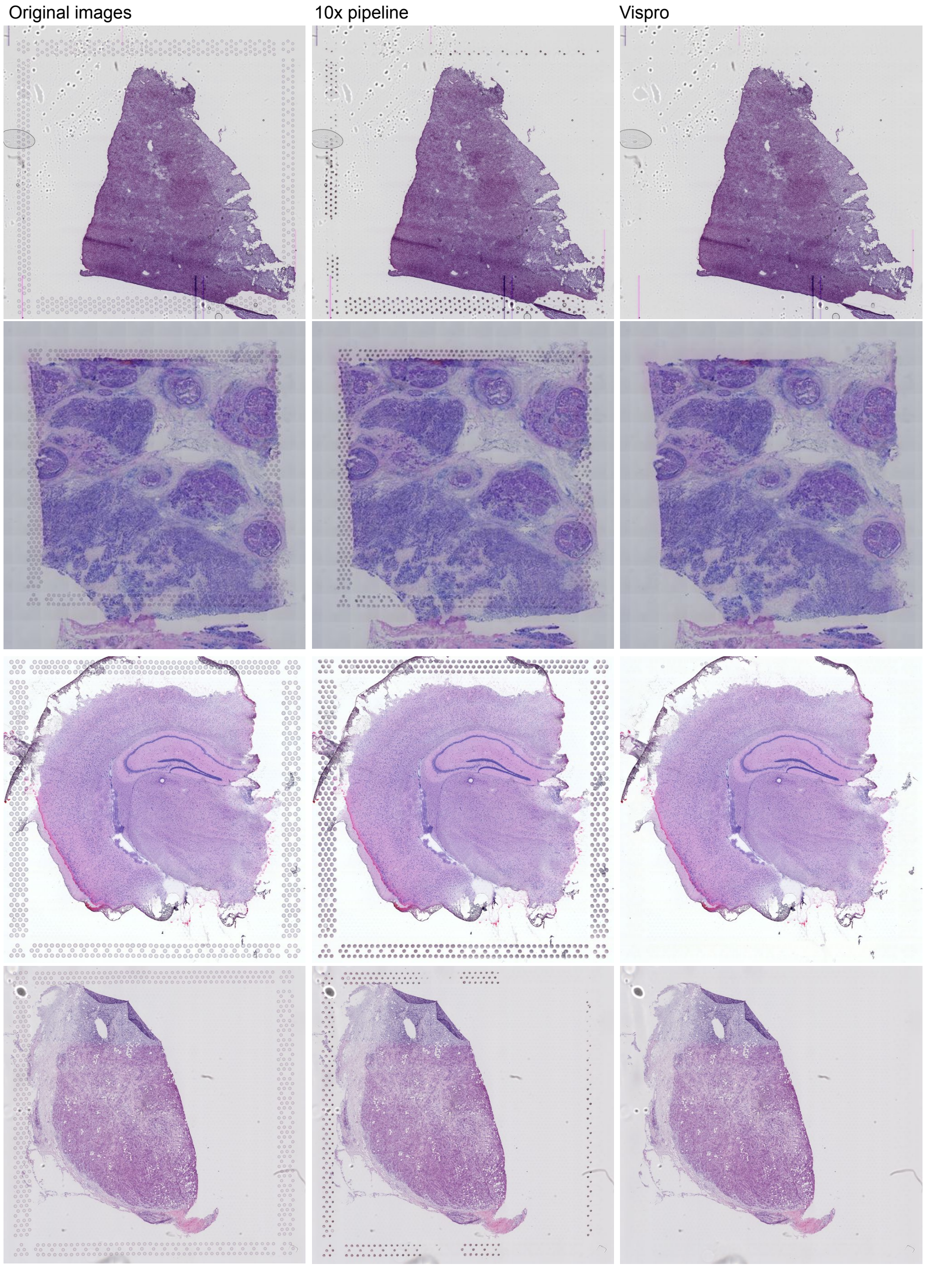

Fig. S1 Additional image restoration results. The three columns display, from left to right: the original images, the restored images with fiducial markers identified by the 10x pipeline, and the restored images with fiducial markers identified by Vispro. Each row represents an image from a Visium sample.

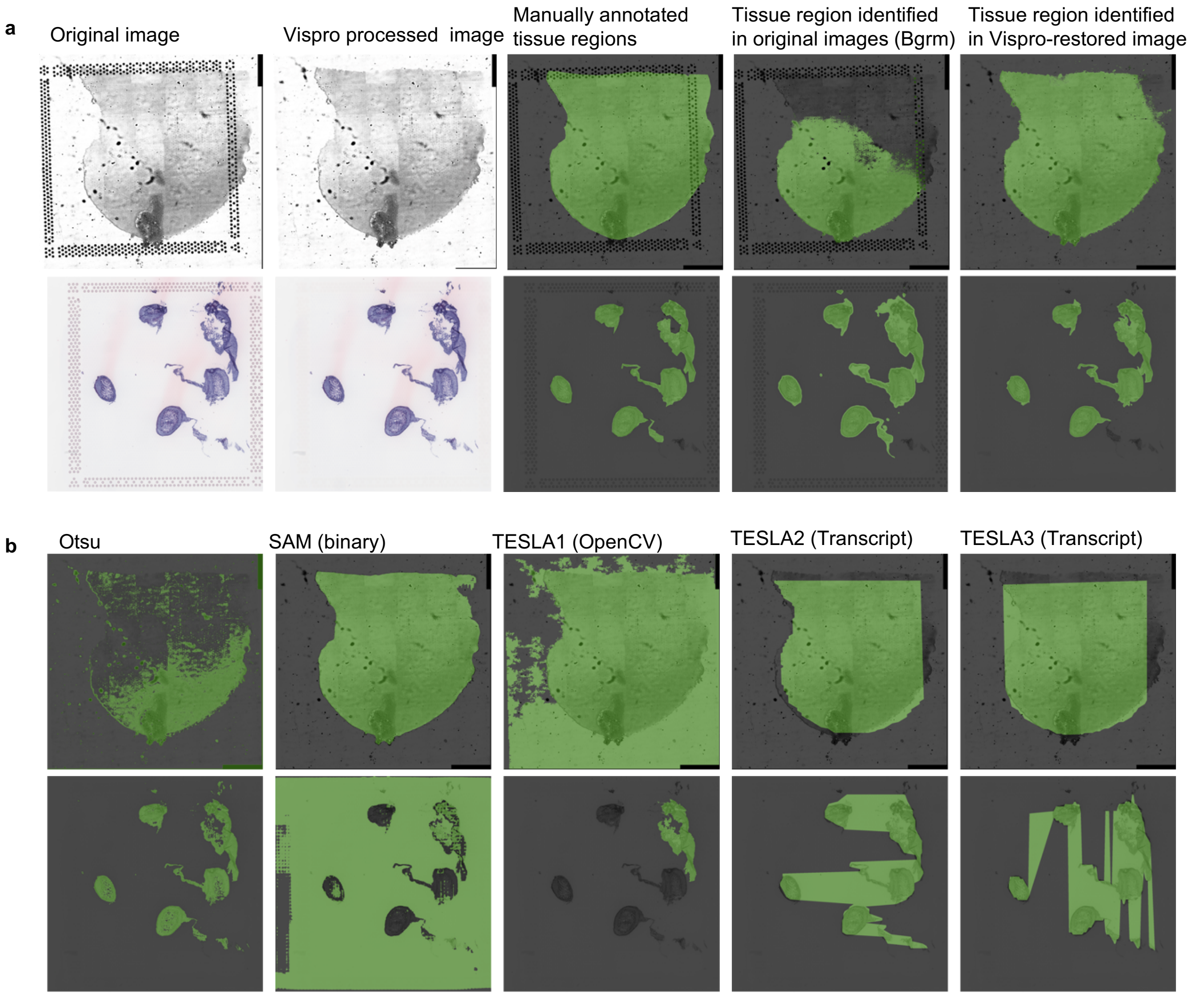

Fig. S2 Additional tissue detection results. **a**, From left to right: the original images, the images processed by Vispro's fiducial marker detection and image restoration modules, the manually annotated tissue areas serving as the gold standard (green regions), the detected tissue regions from the original image (green regions), and the detected tissue regions from the Vispro-processed image in the second column. **b**, Tissue regions identified by competing methods, including Otsu, SAM, OpenCV-based TESLA1, and transcript-based TESLA2 and TESLA3. All methods were applied to images processed by Vispro's fiducial marker detection and image restoration modules.

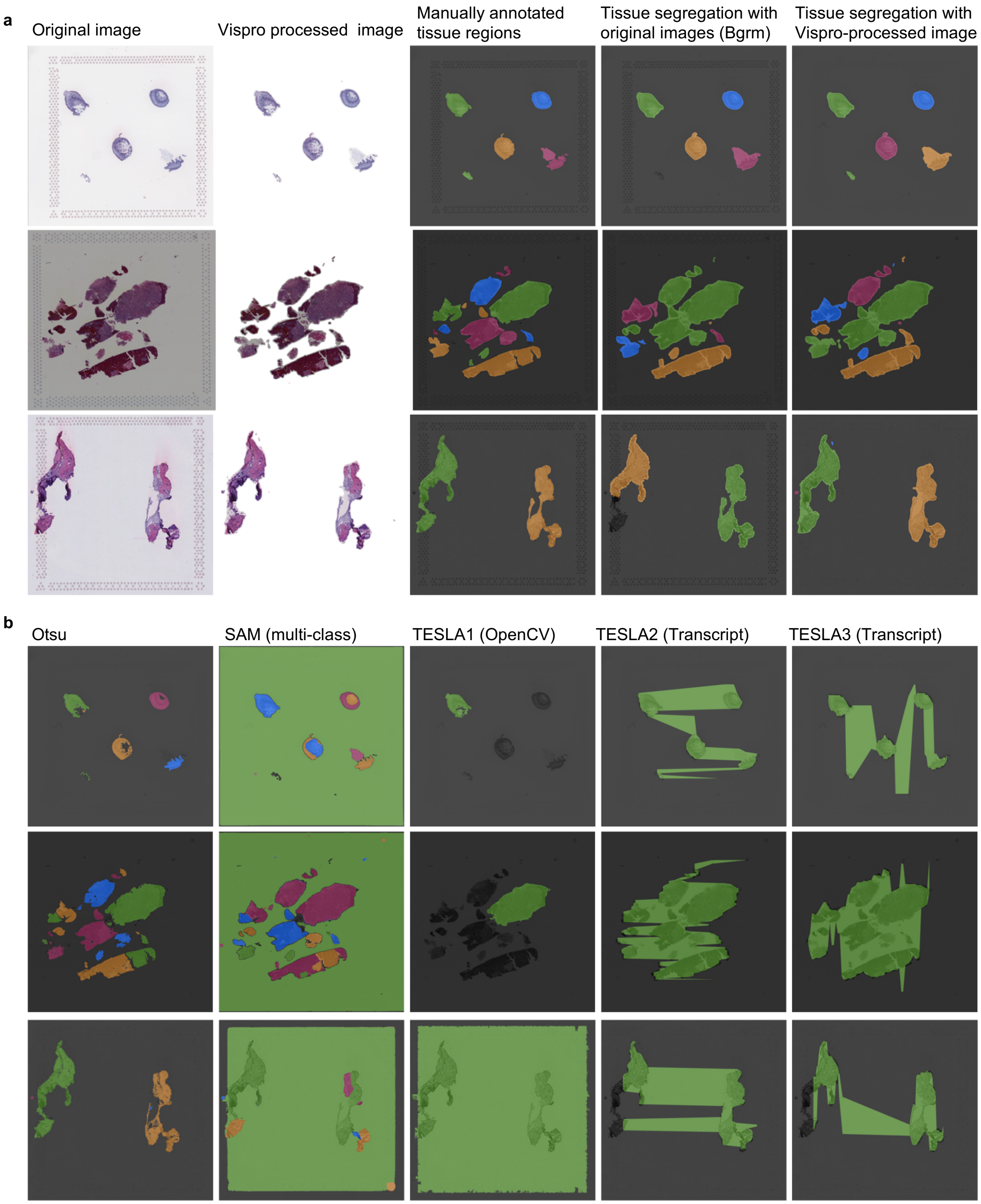

Fig. S3 Additional disconnected tissue segregation results. **a**, From left to right: the original images, Vispro-processed images after tissue detection, manually annotated tissue regions, tissue segregation results using the original image, and tissue segregation results using the Vispro-processed images in the second column. **b**, Tissue segregation results from competing methods on images processed by Vispro's tissue detection module.

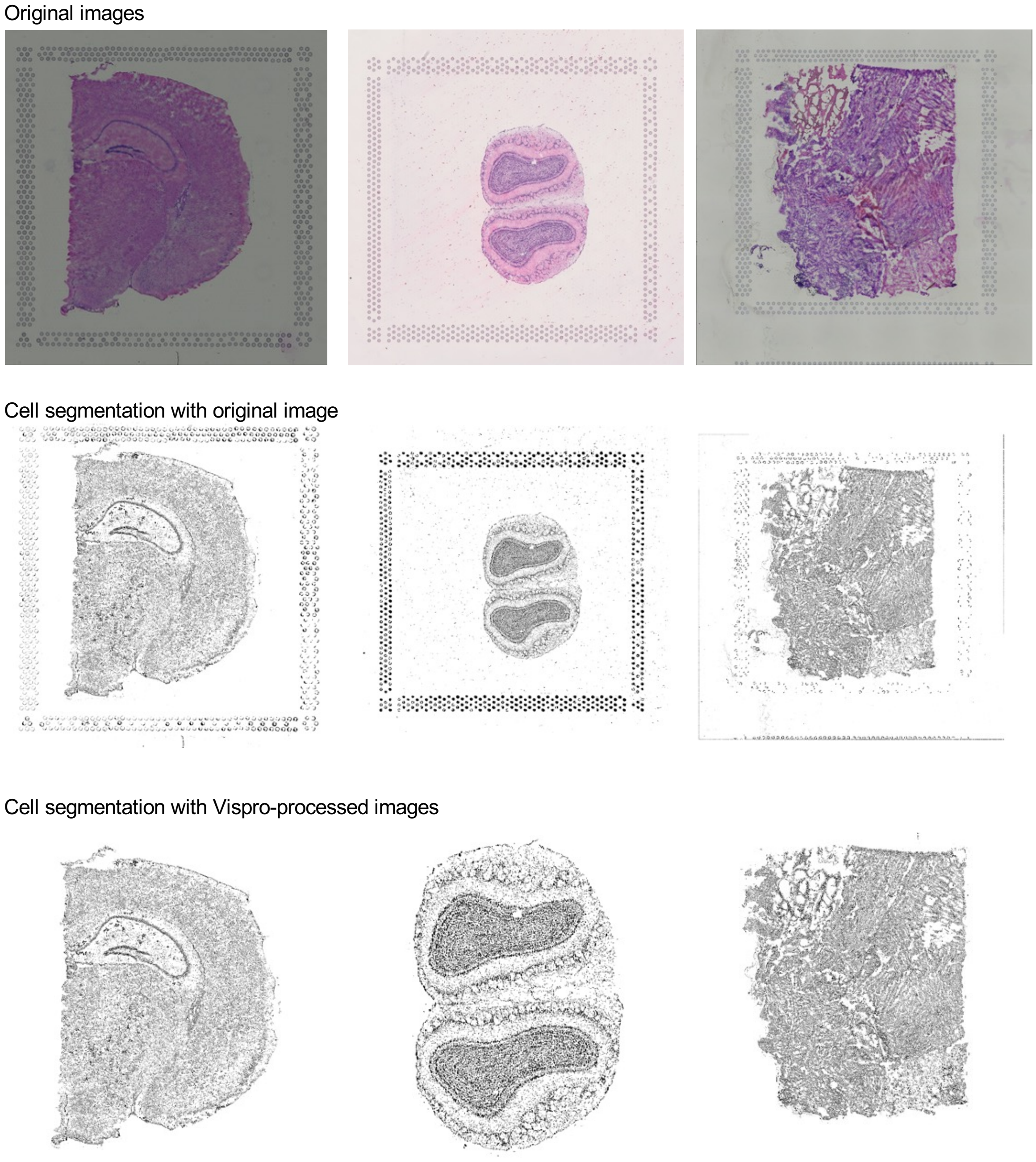

Fig. S4 Additional cell segmentation results. The three rows display, from top to bottom: the original images, cell segmentation results with the original images, and cell segmentation results with the Vispro-processed images. Each column represents an image from a Visium sample.

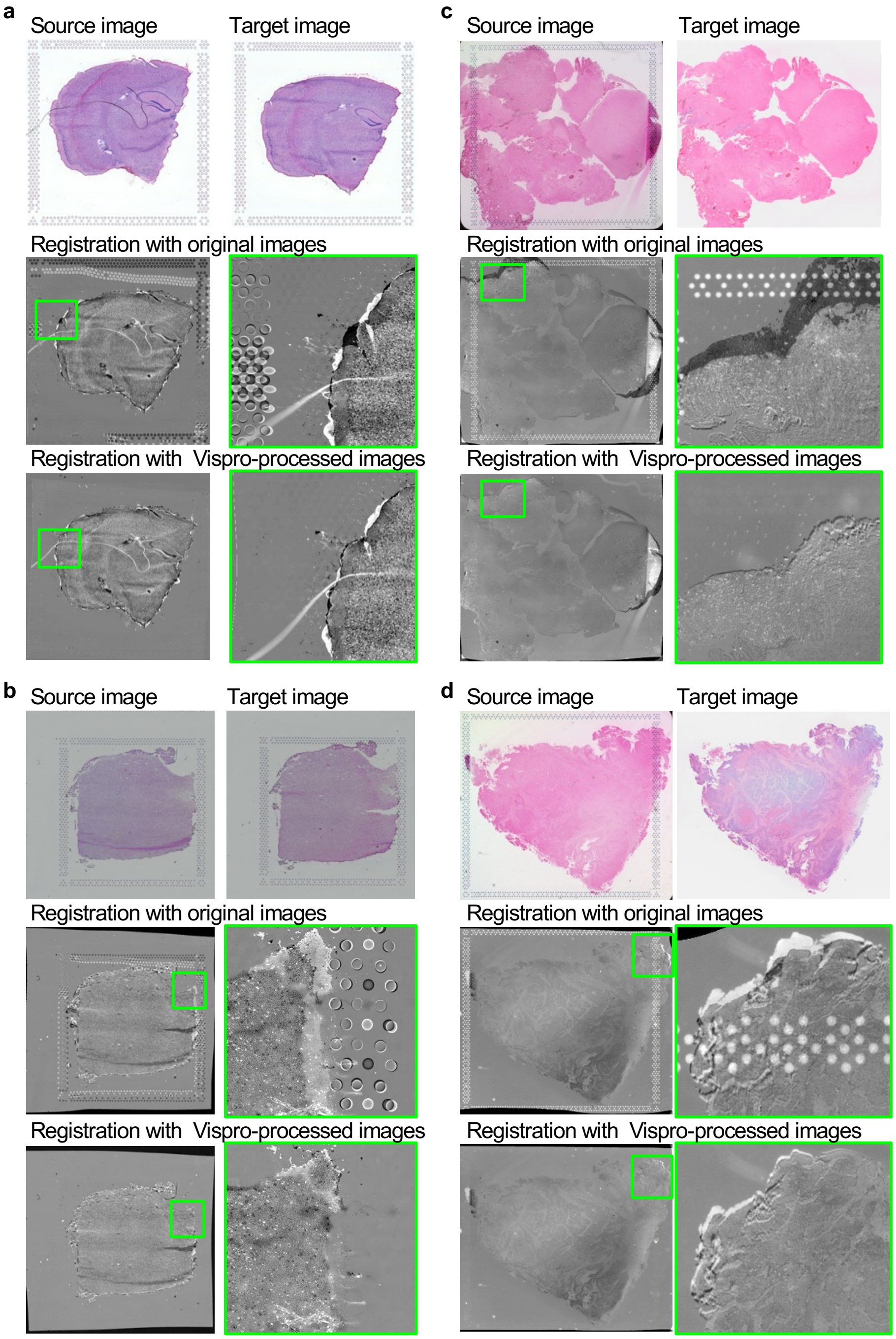

Fig. S5 Additional image registration results. In **a-b**, both the source and target images were generated by Visium. In **c-d** , the source image was generated by Visium, while the target image was generated by the standard H&E workflow. For each panel, from top to bottom: the source and target images, image registration results using the original images, and image registration results using Vispro-processed images. Image registration results display the overlay of the warped source image and the target image in grayscale. The source image is presented with inverted intensity values, while the target image is shown with normal intensity values. Differences between the two images are highlighted as regions of high contrast, appearing as intensely white or black areas.

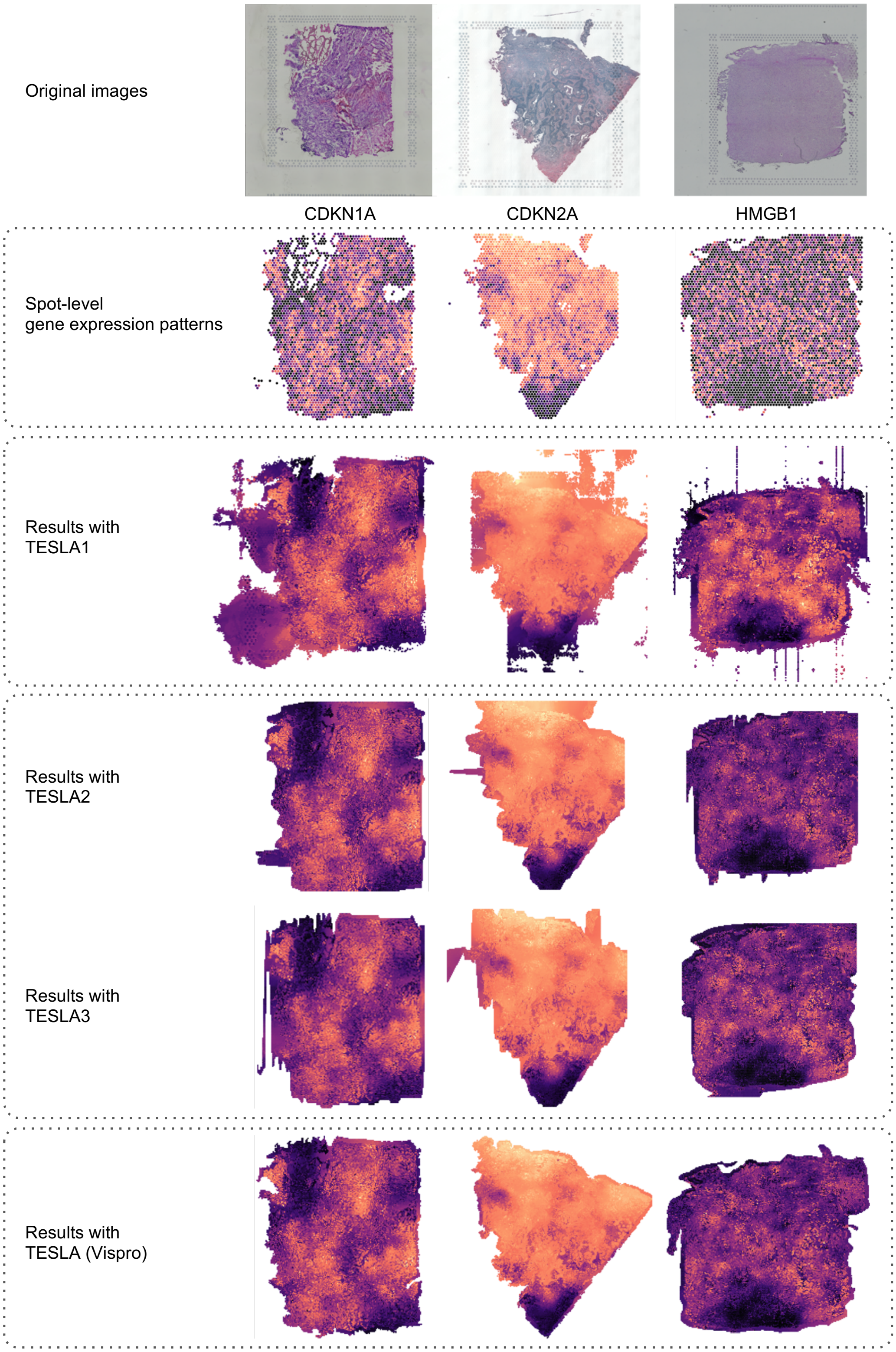

Fig. S6 Additional image-based gene imputation results. The six rows display, from top to bottom: the original images, spot-level spatial gene expression patterns, results with TESLA1 (canny contour detection), results with TESLA2 (scanning transcript contour by spot x), results with TESLA3 (scanning transcript contour by spot y), and results with TESLA (Vispro). Each column represents an image from a Visium sample.

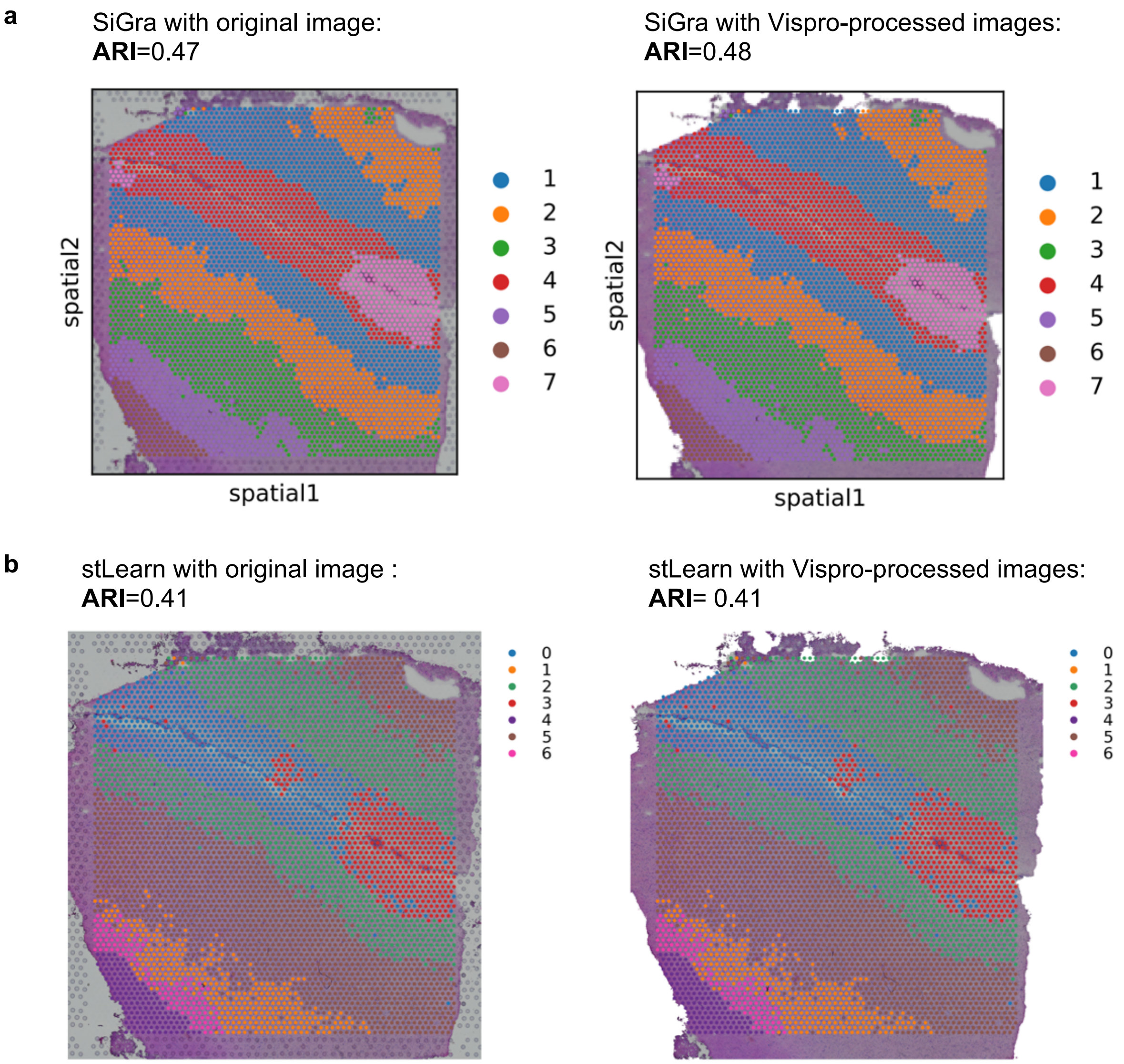

Fig. S7 Spatial domain detection using SiGra and stLearn on original and Vispro-processed images. **a.** Spatial domain detection by SiGra on original (left) and Vispro-processed (right) images. **b.** Spatial domain detection by stLearn on original (left) and Vispro-processed (right) images.
